# Promoting Recovery from Disasters, Pandemics, and Trauma: A Systematic Review of Brief Psychological Interventions to Reduce Distress in Adults, Children, and Adolescents

**DOI:** 10.3390/ijerph20075339

**Published:** 2023-03-30

**Authors:** Annett Lotzin, Alicia Franc de Pommereau, Isabelle Laskowsky

**Affiliations:** 1 Institute for Clinical Psychology and Psychotherapy, Department of Psychology, MSH Medical School Hamburg, 20457 Hamburg, Germany; 2 Department of Psychiatry and Psychotherapy, University Medical Center Hamburg-Eppendorf, 20251 Hamburg, Germany

**Keywords:** disaster, man-made disaster, natural hazard, pandemic, COVID-19, trauma, indicated prevention, low-intensity intervention, brief intervention, subclinical symptoms, psychological distress

## Abstract

A substantial number of survivors of disasters, pandemics, and other severe stressors develop persistent distress that impairs mental health and well-being. However, only a few brief psychological interventions target distress or subclinical symptoms. This systematic review aimed to identify and describe brief psychological interventions to reduce distress or subclinical symptoms in survivors of disasters, pandemics, and other severe stressors. Based on a systematic literature search (MEDLINE, PsycINFO, PSYNDEX, PTSDpubs, and Web of Science), we reviewed published studies and study protocols on self-help, psychosocial support, or brief psychotherapeutic interventions to reduce distress and/or subclinical symptoms following natural hazards and man-made disasters, pandemics, or other traumatic events. We included 27 published studies or study protocols (*n* = 15 RCTs, *n* = 3 controlled pre–post studies, and *n* = 9 uncontrolled pre–post studies) describing 22 interventions. We found evidence for reducing psychological distress and/or subclinical symptoms in 9 out of 15 RCTs, 2 out of 3 controlled pre–post studies, and 9 out of 9 uncontrolled pre–post studies. One RCT provided evidence of increasing well-being. Innovative brief interventions have been developed to reduce distress and/or subclinical symptoms that have an emerging evidence base.

## 1. Introduction

Natural hazards and man-made disasters (e.g., floods and mass violence; [1]), pandemics (e.g., the COVID-19 pandemic; [2]), but also other traumatic events (e.g., train accidents; [3]) can lead to psychological harm. Children are a group particularly vulnerable to suffering the consequences of crises [4]. Most mental health problems following such severe stressors are of a subclinical severity, e.g., [5]. Consequently, there is a strong need for interventions that focus on reducing subclinical symptoms in individuals who have been exposed to a particular stressor but lack a formal mental health diagnosis [6].

Brief evidence-based interventions might be useful to promote recovery and prevent mental disorders. For example, psychological first aid (PFA) is widely known as an evidence-based approach to help individuals and communities cope in the immediate aftermath of a traumatic event [7]. Furthermore, interventions based on cognitive behavioral therapy (CBT) have been found to be effective in reducing distress and subclinical symptoms in individuals who have experienced traumatic events, e.g., [8,9]. The COVID-19 pandemic, which is an acute global challenge and has been shown to have a major impact on psychological stress levels, e.g., [10,11], also highlights the need for mental health treatments. For example, a study conducted in China in 2020 found that 53.8% of respondents rated the psychological impact of the outbreak as moderate or severe [12]. Therefore, we chose to also include COVID-19-related studies within this systematic review.

However, developing and evaluating brief interventions to target subclinical distress after disaster and trauma is a relatively new research field. A couple of narrative or systematic reviews summarized brief interventions on the specific subtypes of disasters, e.g., mental health impacts in response to climate change [13] or the Indian Ocean Tsunami in 2004 [14], or were narrowed to particular age groups, e.g., children and adolescents [15]. There are also a number of reviews that summarize interventions for individuals with a diagnosed mental health disorder, such as PTSD, e.g., [16,17]. A comprehensive systematic review of studies describing or evaluating brief interventions to reduce psychological distress and/or subclinical symptoms in disaster-, pandemic-, or trauma-exposed individuals has not been conducted.

The aims of this systematic review were to (1) systematically identify brief interventions to reduce psychological distress and/or subclinical symptoms in survivors of disasters, pandemics, and other traumatic events and (2) describe their effectiveness.

## 2. Materials and Methods

Our report of this systematic review followed the ‘Preferred Reporting Items for Systematic Reviews and Meta-Analyses’ reporting guidelines, PRISMA; [18]. The review protocol was not published on PROSPERO.

### 2.1. Eligibility Criteria for Study Inclusion

We included published peer-reviewed studies or study protocols in the German or English language (1) that evaluated or planned to evaluate brief psychosocial support, self-help, or brief (≤12 sessions) psychotherapeutic interventions for survivors of disasters, pandemics, and other traumatic events (2) that described or examined interventions to reduce psychological distress, subclinical symptoms of any type of mental disorder, or symptoms of adjustment disorder. To provide an overview not only of completed studies but also of planned or ongoing studies of brief interventions, we included study protocols in this review.

We excluded studies or study protocols that (1) aimed to treat a mental disorder except for adjustment disorder, and studies (2) published before 2010.

### 2.2. Data Sources

We performed a systematic literature search in five databases (MEDLINE, PsycINFO, PSYNDEX, PTSDpubs, and Web of Science) from 1 January 2010 to 1 October 2021. Additionally, we identified studies by hand searching reference lists and contacting researchers.

### 2.3. Search Strategy

The search strategy was based on the PICOS (participants, interventions, comparisons, outcomes, and study design) approach [19]: Section S1 Populations: Disaster-, pandemic-, or trauma-exposed (e.g., term/pandemics); S2 Interventions: Brief interventions (e.g., indicated or low-intensity or brief or behavioral or short-term or low-threshold).mp. adj2 (therap* or treat* or intervention* or modification or train* or program*.ti,ab.); S3 Outcomes: Mental health outcomes relevant for disaster, pandemic, and trauma (e.g., psychological distress OR subclinical symptoms OR adjustment disorder* OR depress* or anxiet* or psychosocial dysfunction*.ti,ab.); S4 Study design: Feasibility or pilot studies, pre–post studies, controlled studies, randomized controlled trials (e.g., Clinical trials/).

### 2.4. Selection Process

Two reviewers (IL and AP) independently screened the titles and abstracts following the inclusion and exclusion criteria. The characteristics of the published studies or study protocols were extracted in an excel table (i.e., authors, year, title, journal, inclusion and exclusion criteria, and the final decision for eligibility). When disagreements occurred between the two reviewers, a third reviewer (AL) was consulted to decide together to include or exclude the study or protocol.

### 2.5. Data Collection Process and Synthesis

Data from the included studies were extracted independently by two reviewers (IL and AP) in an excel sheet. When disagreements in the extracted data occurred between the two reviewers, a third reviewer (AL) was consulted. The following data were extracted: study, intervention name and description, study design, timepoint of intervention after stressor exposure (e.g., 60 days or more), number of sessions, type of stressor, target population, delivery format, assessed outcomes, and results (see Appendix A).

## 3. Results

### 3.1. Study Selection

**Figure 1:**
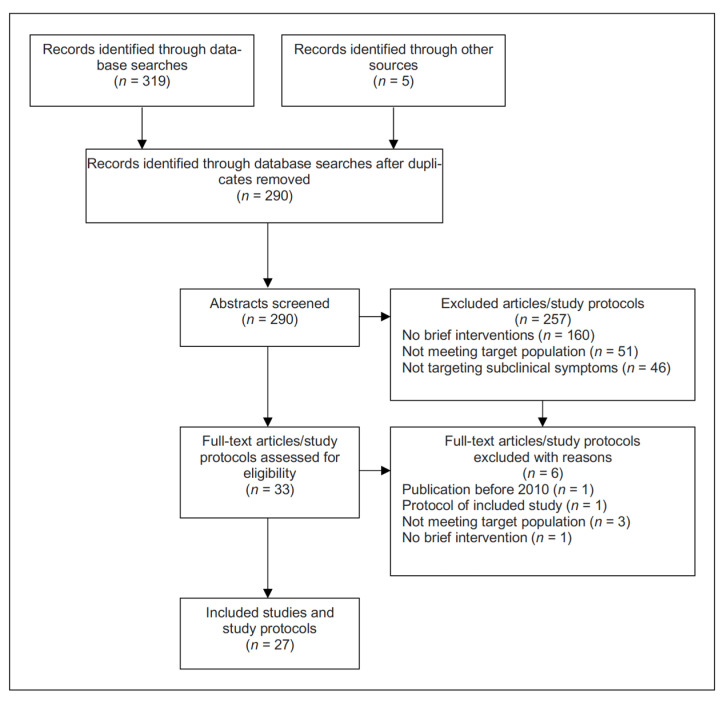
Flowchart of the review process.

Database searches identified *n* = 319 records; 5 additional records were found through searching reference lists and contacting researchers (Figure 1). After removing 34 duplicates, screening abstracts, and reviewing full articles and study protocols, 21 studies and 6 study protocols were included that reported on brief interventions to reduce distress and/or subclinical symptoms in survivors of disasters, pandemics, or trauma.

### 3.2. Study Characteristics

The study characteristics are listed in Table 1. The 27 studies or study protocols were from 16 countries; most were from America or Asia. More than half of the described studies (*n* = 15; 55.6%) were RCTs; nine studies (33.3%) used an uncontrolled pre–post study design, and three studies (11.1%) were designed as a controlled pre–post study. Out of the 27 articles/study protocols, 11 articles/study protocols (40.7%) described studies on the survivors of natural hazards, 3 (11.1%) described studies on the survivors of man-made disasters, 8 (29.6%) described studies on the survivors of pandemics, and 5 (18.5%) described studies on survivors of other types of trauma. Twelve (44.4%) articles/protocols described studies examining the efficacy of psychosocial support interventions; nine (33.3%) articles/protocols described studies on the efficacy of brief psychotherapeutic treatments; and six (22.2%) articles/protocols reported on studies of self-help interventions.

## 4. Intervention Characteristics and Study Results

The characteristics of the brief interventions described in the included articles/protocols are summarized in Table A1 of Appendix A. More detailed information on intervention characteristics and results (e.g., effect sizes) of the included studies/protocols are provided in Table A2 of Appendix A.

### 4.1. Self-Help Programs

Five self-help programs targeted adults, and one intervention focused on adolescents and their parents. The interventions were mostly conducted online; only one intervention was designed in a hybrid format. Four of the six self-help interventions were developed in a pandemic context, and the remaining two interventions were designed to be applied after disasters. Three of the six interventions comprised additional psychotherapeutic or psychological support.

#### 4.1.1. Pandemic-Focused Interventions

Computerized CBT (cCBT) [20] is a one-week online self-help intervention based on cognitive behavioral therapy (CBT). cCBT aims to reduce acute psychological distress and symptoms of depression, anxiety, and insomnia in adults during the COVID-19 pandemic. Three modules cover cognitive training, cognitive consolidation, and behavioral interventions. The intervention was tested in an RCT during the COVID-19 pandemic in China with *N* = 252 adult COVID-19-infected patients [20]. A significant decrease in depressive and anxiety symptoms was found at one-month follow-up in the cCBT + treatment as usual (TAU) group compared to a TAU-only group (psychological assessments, psychological support, consultations about well-being, and COVID-19).

The Individualized Short-term Training Program [21] is a self-help program that is combined with psychological support targeting depression and anxiety symptoms of emergency nurses during the COVID-19 pandemic. The program includes online and face-to-face elements. The length of the intervention and the number of sessions were not reported by the authors. The intervention covers knowledge about diagnosing COVID-19, handling and safety precautions with infected patients, and psychological support including mindfulness-based stress reduction. Psychologists provide online and practical face-to-face training and psychological support to nursing staff. The self-help online training is delivered asynchronously through videos, graphics, and texts. In an uncontrolled pre–post study, the authors of the intervention evaluated the Individualized Short-term Training Program in *N* = 71 female Chinese nurses working in an emergency isolation department during the COVID-19 pandemic [21]. A significant anxiety reduction but not in depression was found post-training.

Online Psychotherapy Tool (OPTT) [22] is a 9-week program to reduce mental health problems in adults during the COVID-19 pandemic. OPTT consists of a self-help module and psychotherapeutic support, with the main focus on self-help. The intervention combines CBT, mindfulness therapy, and problem-based therapy. Weekly 40 min self-guided web-based online modules are available, participants can interact with their therapist through a chat function within the online platform, and a therapist provides individual written feedback. The authors plan to conduct a nonrandomized controlled trial with *N* = 80 Canadian adults with anxiety symptoms during the COVID-19 pandemic [22]. The effects of the intervention on anxiety, depressive symptoms, resilience, and quality of life will be compared to a TAU control group. No study results have been reported yet.

“My Health too” [23] is a seven-session CBT-based online self-help program with an option for psychotherapeutic support (i.e., the possibility to call a CBT-trained psychologist). The program was designed for health care workers to reduce psychological distress during the COVID-19 pandemic and to prevent its long-term consequences. A total of 7 20 min asynchronous (i.e., the interaction does not happen in real time) video sessions cover psychoeducation on stressors, adaptive behavioral and cognitive coping strategies, mindfulness and acceptance of stressors, promoting action toward values, addressing barriers and motivation, and self-compassion. Weiner et al. [23] is currently investigating whether the intervention reduces distress in *N* = 120 French healthcare workers during the COVID-19 pandemic. In an RCT, the intervention group is compared to an active control group receiving bibliotherapy. No results have been published to date.

#### 4.1.2. Disaster-Focused Interventions

The web-based Bounce Back Now (BBN) [24] is a four-session self-help program for disaster-affected adolescents and parents aiming to reduce post-disaster mental and behavioral problems. Four modules provide skills and strategies to cope with stress- and trauma-related symptoms, smoking, and alcohol use, and mood-related symptoms. The stress and trauma module provides education about PTSD symptoms and evidence-based trauma-focused interventions, a reduction in the avoidance of traumatic cues, coping strategies, and anxiety management. An additional self-help intervention for adults (Adult Self-Help, ASH) can be added to BBN, BBN + ASH [24]. A pre–post-test study with *N* = 979 US adolescents affected by tornadoes in Missouri and Alabama in 2011 [25] revealed a significant decline in PTSD symptoms in the BBN condition at 4- and 12-month follow-up compared to the control condition in which no education or recommendations were offered. Instead, participants were given quizzes and questions about myths and facts.

The Disaster Recovery Web (DRW) Project [26] is a self-help web-based program for adults affected by natural hazards to reduce symptoms of PTSD, depression, and anxiety. It is applied in the acute aftermath of a disaster and consists of four web modules educating about post-traumatic stress, depressed mood, generalized anxiety, and panic. There is no interaction with a therapist. A pre–post-test study without a control group conducted after Hurricane Ike in Texas with *N* = 1249 adult survivors [27] found no significant reduction in depressive symptoms or PTSD symptoms 4-months post-intervention.

### 4.2. Psychosocial Support Programs

Five psychosocial support programs targeted adults, and one intervention was developed for children and their parents. Two of the six psychosocial support programs were pandemic-focused, one intervention was disaster-focused, and three interventions targeted other types of trauma. Four interventions were designed as face-to-face interventions, although two of them could be applied either in a face-to-face or online format. The remaining two interventions were designed as online or phone-based interventions.

#### 4.2.1. Pandemic-Focused Interventions

Grief Counseling for Adults [28] is a psychosocial support program to promote better life adaptation after loss. The program was developed to support bereaved Chinese people during COVID-19. A trained counselor delivers 8 to 10 online sessions lasting 1 h. Grief counseling for adults is based on current grief treatment approaches, including meaning reconstruction [29]; complicated grief treatment, CGT; [30], and cognitive behavioral therapy for complicated grief, CBT-CG; [31]. It covers understanding and managing grief reactions; managing painful emotions; learning self-care; increasing contact with others; coping with difficult days; and adapting to a new life. Grief counseling for adults will be evaluated in a single-blinded RCT among *N* = 160 bereaved Chinese adults who have lost their first-degree relative during the COVID-19 pandemic [28]. The researchers aim to evaluate the effects of the intervention on prolonged grief symptoms, PTSD, and depression at baseline, post-intervention, and at 3-month follow-up relative to a wait list control group. The results of this study have not yet been published.

Resiliency Engagement and Care in Health (REaCH) [32] is a 4-week psychosocial intervention for people with socioeconomic vulnerability during the COVID-19 pandemic. REaCH targets mental well-being, depressive symptoms, and perceived social support by providing proactive engagement and crisis intervention, problem-solving-oriented support therapy, and assertive linkage with community resources. It involves a synchronous telephonic befriending program consisting of 4 phone calls, each 0.5 to 1 h, delivered by lay workers and nonhealth professionals. The authors plan a cluster-randomized controlled trial (cRCT) to examine the REaCH intervention with *N* = 1440 economically disadvantaged and vulnerable Indian adults during the COVID-19 pandemic [32]. The intervention group will be compared with a control group receiving four phone calls informing about COVID-19.

#### 4.2.2. Disaster-Focused Interventions

The Mental Health Integrated Disaster Preparedness (MHIDP) intervention [33] is aimed at improving disaster preparedness, reducing mental health symptoms, and fostering community cohesion in adults affected by natural hazards in Haiti. MHIDP includes establishing safety and practicing skills to cope with disaster-related distress, the provision of space for sharing personal experiences, and training in disaster preparedness (e.g., creating preparedness kits comprised of basic supplies). The content is provided within 3 sessions lasting 6 h. MHIDP intervention was evaluated in an RCT among *N* = 480 adults exposed to earthquakes and floods in Haiti [33]. The program was delivered by 2 trained lay mental health workers in groups of up to 20 participants. The intervention group was compared to a wait list control group across three timepoints (i.e., baseline, 3–4-month, and 7–8-month follow-up). Disaster preparedness behavior significantly increased among intervention participants, while depressive, anxiety, and PTSD symptoms significantly decreased at both follow-ups. A significant reduction in functional impairment was evident at 3–4-month follow-up but disappeared at 7–8-month follow-up.

#### 4.2.3. Interventions Focusing on Other Severe Stressors

Problem Management Plus (PM+) [34] is a brief five-session program to reduce distress in adults living in communities affected by adversity or crises. PM+ includes managing stress, managing problems, behavioral activation (i.e., get going and keep doing), and strengthening social support. Trained nonspecialist lay providers deliver the intervention. PM+ was tested in a cRCT in *N* = 121 participants [35] in earthquake-affected communities in Nepal. Participants in the treatment group received five sessions of PM+ in a group setting, and participants in the control group received enhanced TAU (which entailed brief psychoeducation and the provision of referral options to primary care services). Depressive symptoms, daily functioning, psychological distress, PTSD symptoms, and psychosocial problems improved more in the PM+ arm than the enhanced TAU arm at 8 weeks post-intervention. A cRCT [36] investigated an adapted PM+ program with six to eight participants per group, which was compared to enhanced usual care (psychoeducation and a referral option to primary care providers trained in mental healthcare). *N* = 611 adults from disaster-prone regions in Nepal received 5 weekly sessions of approximately 2.5 h. The PM+ group showed lower psychological distress and depression symptoms, and had fewer “heart-mind” problems compared to the control group at 3-months post-treatment. However, the PM+ group did not show an improvement in functional impairment and PTSD symptoms. An adapted version of PM+ for participants living in conflict-affected Peshawar in Pakistan was tested in a pilot RCT [37] in *N* = 60 participants, compared to enhanced TAU (mental health care management by trained general care practitioners). Functioning and PTSD symptoms improved more in the PM+ group post-intervention, but no significant changes in psychological distress could be observed between the groups. The effects of PM+ were compared to facility-based enhanced TAU provided by community nurses in an RCT in *N* = 421 Kenyan women exposed to physical or sexual abuse [38]. The PM+ group showed significant improvements in psychological distress, daily functioning, PTSD symptoms, and personally identified problems in the change from baseline to 3-month follow-up. An RCT [39] is planned to examine an adapted version of videoconferencing PM+ in *N* = 240 adults in a group context of 3–4 participants in Sydney, Australia. The intervention will be delivered over 6 weekly 60 min sessions and will specifically target COVID-19-related distress. The control group will receive enhanced TAU (emailed handouts with PM+ strategies and no expert assistance). In addition to psychological distress, rumination, sleep problems, anhedonia, social support, and COVID-19-related stress will be examined.

The Skills fOr Life Adjustment and Resilience program (SOLAR) [6] is a brief five-session psychosocial support program to reduce persistent distress or subclinical symptoms in adults impacted by a disaster or trauma. It is delivered by trained coaches that can be non-mental-health professionals. SOLAR covers six modules: healthy living, managing strong emotions, getting back into life, coming to terms with the disaster, managing worry and rumination, and maintaining healthy relationships. An uncontrolled pre–post 3-month follow-up pilot study in *N* = 15 Australian bushfire survivors [6] demonstrated reductions in psychological distress, post-traumatic stress symptoms, and functional impairment at post-treatment. Another controlled pre–post pilot study [40] proved the acceptability, feasibility, and efficacy of a culturally adapted version of SOLAR with *N* = 99 Pacific Islanders that were exposed to Tropical Cyclone Pam in 2015. SOLAR was administered in a group format of up to 10 participants compared to a TAU control group, which included informal familial, community, and church-based support. Reductions in psychological distress, PTSD symptoms, and functional impairment were found in the intervention group relative to the control group from pre- to post-intervention. The SOLAR group program was evaluated in a randomized controlled feasibility study in *N* = 30 German survivors of different types of trauma [41]. Participants in the SOLAR group intervention showed a greater reduction in psychological distress, symptoms of insomnia, patient-centered outcomes, functional impairment, quality of life, and perceived social support post-intervention, compared to a wait list control group. Symptoms of PTSD did not decrease more greatly in the intervention group relative to the control group.

Listen Protect Connect (LPC) [42] is a school-based PFA program for children. LPC provides basic psychological support and aims to reduce the initial distress of students and parents following traumatic events, such as community disasters, emergencies, or personal trauma. LPC is delivered by non-mental-health professionals. It is based on the five-step crisis response strategy “Listen, Protect, Connect—Model & Teach” [42]. An adapted version of LPC (composed of three steps: listen, protect, and connect) was piloted in an uncontrolled pre–post study in *N* = 20 US-American children impacted by the Great Flood of Iowa in 2008 to reduce PTSD symptoms [43]. The school nurse provided 1 on average 25 min LPC session to each student. Depressive symptoms and felt connectedness to their school improved at 4 weeks post-intervention. Perceived social support increased at 8 weeks post-intervention. PTSD symptoms (i.e., re-experiencing, avoidance, and arousal) did not significantly decrease at 8 weeks post-intervention.

### 4.3. Brief Psychotherapeutic Programs

We identified ten brief psychotherapeutic interventions, of which four were developed for children or adolescents. Two brief psychotherapeutic interventions were disaster-focused; the remaining eight interventions addressed survivors of other types of trauma, although some of these interventions could also be applied in the aftermath of natural hazards and man-made disasters. There were no interventions specifically designed for use during a pandemic. All interventions were designed as face-to-face interventions.

#### 4.3.1. Disaster-Focused Interventions

The Brief School-Based Cognitive Behavioral Intervention [44] is a one-session psychotherapeutic intervention for disaster-affected adolescents to reduce PTSD and depressive symptoms. The intervention is based on the cognitive behavioral model of post-traumatic stress disorder [45] and consists of a single 90 min session, delivered by trained CBT clinical psychologists. It involves four steps: the identification of problems, psychoeducation, decreasing negative appraisal, and the practice of relaxation breathing. A pilot study [44] examined the intervention in the context of pre-, post-, and follow-up measurements without a control group. *N* = 22 adolescents affected by the Great East Japan Earthquake in 2011 were divided into 2 groups, each with 11 adolescents. The results showed significant improvements in PTSD symptoms post-intervention which were maintained at a 4-month follow-up. There was no significant reduction in depressive symptoms.

Strength after Trauma (StArT) [46] is a brief manual-based trauma-focused CBT intervention for disaster-exposed adolescents to reduce PTSD symptoms. StArT compromises five modules: psychoeducation, cognitive restructuring, exposure, problem solving, and relapse prevention. It includes 10 sessions of 1 h and is delivered by a psychotherapist. StArT was piloted in an uncontrolled pre–post study in *N* = 6 American children exposed to Hurricane Katrina in 2005 [47]. The results suggest that the 10-session intervention was feasible when conducted in a school setting. Negative cognitions and PTSD symptoms significantly declined between pre- and post-treatment. No significant reduction in anxiety symptoms could be observed.

#### 4.3.2. Interventions Focusing on Other Severe Stressors

Cognitive Behavioral Therapy for Post-disaster Distress (CBT-PD) [48] is an 8- to 12-session CBT intervention for adults impacted by major disasters, terrorism, or traumatic events to reduce post-disaster distress. The intervention is delivered by trained therapists. CBT-PD includes psychoeducation, coping skills, and cognitive restructuring. Coping skills include breathing retraining and behavioral activation. In an uncontrolled pre–post-test study, *N* = 342 adults from New York State, US, exposed to Hurricane Sandy in 2012 [49], were assessed at referral, baseline, intermediate treatment, as well as at post-treatment and 5-month follow-up. Significant reductions in distress throughout the intervention were found, with large improvements from pre- to post-treatment.

Exposure-based Cognitive Behavioral Therapy for children [50] aims to reduce PTSD symptoms in children in 4–8 60 min sessions with possible parent support. The intervention includes five elements: psychoeducation, repeated exposure to the trauma memory, cognitive restructuring, exploring and correcting undesired or unhelpful coping behavior, and relapse prevention. Eye Movement Desensitization and Reprocessing (EMDR) based on Shapiro [51] is typically delivered in 6–12 sessions to reduce PTSD symptoms in children, adolescents, and adults. EMDR applies an eight-phase approach: (phase 1) history taking, (phase 2) preparing the client, (phase 3) assessing the target memory, (phase 4–7) processing the memory to adaptive resolution, and (phase 8) evaluating treatment results. De Roos et al. [52] conducted an RCT comparing exposure-based CBT and EMDR in reducing disaster-related PTSD symptoms in *N* = 52 children and adolescents aged from 4 to 18, 6 months after the explosion of a fireworks company in Enschede, Netherlands. Participants with disaster-related clinical symptoms were included in the study. They received up to 4 individual sessions delivered by a clinical psychologist over 4–8 weeks along with up to 4 sessions of parental guidance. Both treatment approaches produced significant reductions in PTSD symptoms, anxiety symptoms, depressive symptoms, and behavioral problems post-intervention. The study did not find significant differences in the outcomes between the groups.

The Preventive Resilience Training for Unaccompanied Refugee Minors [53] is a CBT intervention consisting of 6 90 min sessions to reduce trauma-related symptoms such as PTSD, depression, and anxiety in adolescent refugees with trauma exposure. The resilience training includes psychoeducation, cultural resources, and emotion regulation strategies. The intervention is a group-based program delivered by clinical psychologists or social workers with training in trauma therapy. The authors conducted an RCT in *N* = 55 Australian male adolescent refugees from Afghanistan and Pakistan with flight experience [53]. The intervention group showed an increase in general well-being at 7 weeks post-intervention compared to the wait list control group. However, no reduction in anxiety, PTSD, and depressive symptoms could be found.

Mindfulness-Based Stress Reduction (MBSR) [54] is a transdiagnostic intervention to improve mindfulness. The intervention aims at developing four mindfulness practices (sitting meditation, walking meditation, mindful movement, and a body scan) to influence perceived distress, anxiety, depression, emotion dysregulation, and PTSD symptoms. It consists of 8 weekly 120 min sessions and one 240 min retreat session delivered face-to-face by an experienced MBSR teacher in groups of up to 30 participants [55]. Gallegos et al. [56] piloted MBSR in an uncontrolled pre–post study in *N* = 50 US-American adult women with a history of interpersonal childhood trauma. The results showed a significant reduction in perceived distress, depressive symptoms, anxiety, emotion dysregulation, and PTSD symptoms at post-intervention and 1-month follow-up compared to baseline. Mindfulness also significantly increased.

The Trauma Therapy Program [57] is an intervention comprising 6 sessions of 90 min to reduce work-related distress in health practitioners working in emergency services. The intervention aims to reduce symptoms of anxiety, depression, and PTSD. The program incorporates trauma-focused cognitive behavioral therapy, TF-CBT [58], and eye movement desensitization and reprocessing, EMDR [51]. The intervention is delivered by therapists trained in TF-CBT or EMDR. The program was tested in an uncontrolled pre–post-test study in *N* = 429 emergency service professionals from the UK [57]. The intervention effectively reduced anxiety, depression, and PTSD symptoms post-treatment compared to pre-treatment.

Emotional Freedom Techniques (EFT) [59] is a brief psychotherapeutic intervention to reduce psychological distress and symptoms of depression, anxiety, and PTSD. The intervention has a flexible number of sessions depending on the type and severity of the problems. EFT includes trauma exposure, cognitive, and somatic therapeutic components; it combines the exposure to traumatic memories with self-acceptance statements derived from cognitive therapy while applying psychological acupressure (i.e., tapping) as a stress relief technique. EFT is delivered by EFT-certified therapists. The effect of 6 EFT sessions 60 min was examined in an RCT with *N* = 55 US veterans from Iraq or Afghanistan war [60]. The EFT intervention focused on combat-related traumatic events and was delivered in combination with the TAU of a Veteran Administration (VA) hospital. The results showed significant reductions in PTSD, anxiety, depression, hostility, obsessive–compulsive behavior, paranoia, phobic anxiety, psychoticism, and somatization in the EFT group compared to the TAU group at post-intervention. Improvement in symptoms was maintained until the 3-month and 6-month follow-up. Interpersonal sensitivity did not significantly improve.

Solution Focused Brief Therapy (SFBT) [61] is a brief future- and goal-oriented psychotherapeutic intervention for adolescents and adults which can be applied to a wide range of issues. The intervention, comprising 5 sessions of 45 min., aims to explore current resources and future hopes [62]. An RCT assessed the effectiveness of SFBT in a group of *N* = 76 Chinese adolescents to reduce anxiety symptoms during the COVID-19 pandemic [63]. The intervention group received 2–4 sessions of SFBT via videoconferencing within 2 weeks, whereas the wait list control group received 2–4 sessions of counseling service. It was hypothesized that participants assigned to SFBT would have better clinical outcomes in terms of anxiety symptoms, depressive symptoms, and coping strategies than participants in the control group. The study results have not been published yet.

## 5. Discussion

This systematic review aimed to identify and synthesize brief interventions to reduce distress and/or subclinical symptoms in individuals exposed to disasters, pandemics, or other traumatic events. We considered 27 published articles or study protocols (including 15 RCTs) that reported on 22 different brief interventions to reduce distress and/or subclinical symptoms. Out of the 27 included articles or protocols, 10 were published articles describing intervention studies, and 5 were study protocols.

### 5.1. Self-Help Programs

Six published articles or study protocols described studies on the efficacy of brief self-help interventions. Two articles reported on an RCT or an uncontrolled pre–post study evaluating self-help programs for COVID-19 survivors (cCBT and the Individualized Short-term Training Program). Both studies reported significant reductions in anxiety at one-month follow-up after the application of cCBT [20] and post-training after the application of the Individualized Short-term Training Program [21]. Only the RCT on cCBT found reductions in depressive symptoms post-intervention [20]. Hence, there is the first evidence for two self-help interventions, namely cCBT and individualized short-term training, based on one RCT and one uncontrolled pre–post study, that these self-help programs may reduce anxiety in COVID-19 survivors in the short-term. Two additional study protocols on self-help programs for COVID-19 survivors were identified which might provide further evidence in the future [22,23].

Two published studies evaluated self-help programs (BBN and the DRW Project) for survivors of natural hazards (i.e., tornadoes and hurricanes). One article reported on a controlled pre–post study that found a significant decline in PTSD symptoms in adolescents and their parents at follow-up measurements after the application of BBN [25]; another article reported on an uncontrolled pre–post study that found no evidence for the efficacy of the DRW Project to reduce PTSD or depressive symptoms [27]. The lack of effectiveness of the DRW Project could be attributed to the fact that it was applied by the participants one year after the disaster. This may result in individuals being less motivated to engage with this intervention or having already recovered on their own.

We found no published articles or study protocols on studies evaluating a self-help program for survivors of other types of traumatic events.

### 5.2. Psychosocial Support Programs

Twelve published articles or study protocols described the evaluation of psychosocial support programs. We did not identify any evidence for the efficacy of psychosocial support programs for COVID-19 survivors. Two study protocols have been published [28,32] that describe evaluation studies on psychosocial support programs among COVID-19 survivors, but no results have been published yet.

We identified evidence for one psychosocial support program (MHIDP intervention) targeting survivors of natural hazards (i.e., earthquakes and floods) to improve depressive, anxiety, and PTSD symptoms at follow-up assessments [33].

Nine identified published articles or study protocols described psychosocial support programs originally developed for survivors of other severe stressors, e.g., exposure to community adversity, crises, or interpersonal abuse. However, most of these interventions could be applied to other types of stressors, such as natural hazards. An uncontrolled pre–post study [43] showed that the psychosocial support intervention LPC [42] for children after experiencing traumatic events, such as community disaster, emergency, or personal trauma, was successful in reducing PTSD and depressive symptoms at post-intervention.

We found five published articles or study protocols that described PM+ [34] for communities affected by adversity or crises. Two RCTs on PM+ [37,38] indicated a significant improvement in PTSD symptoms and daily functioning after the provision of PM+ using an individual format. Significant improvements in psychological distress at 3-month follow-up were only observed in the study of Bryant and colleagues [38]. The results of two cRCTs [35,36] indicated that PM+ applied in a group significantly reduced psychological distress and depressive symptoms post-intervention. However, only in the study of Sangraula and colleagues [35], were PTSD symptoms significantly reduced post-intervention. We found one study protocol [39] that has not yet published results. Overall, the results of a few studies indicate that PM+ leads to a significant decline in psychological distress and PTSD symptoms post-treatment. Our systematic literature search yielded three articles or study protocols reporting on studies on SOLAR [6]. A pilot RCT [41] reported significant reductions in psychological distress; PTSD symptoms did not significantly decrease in the SOLAR group relative to the control group, as PTSD symptoms declined in both groups. The results of one uncontrolled [6] and one controlled pre–post study [40] indicated significant reductions in psychological distress and PTSD symptoms. In sum, there is emerging evidence based on three studies that SOLAR leads to a significant decline in psychological distress, and evidence based on two (un)controlled pre–post studies that SOLAR effectively reduces PTSD symptoms.

Overall, in the field of psychosocial support programs, most evidence for their efficacy is currently available for PM+, for which some RCTs have been conducted. SOLAR is another promising psychosocial support program with an emerging evidence base.

### 5.3. Brief Psychotherapeutic Programs

Nine studies examined or planned to examine the efficacy of brief (i.e., max. 12 sessions) psychotherapeutic interventions. No studies described interventions designed for an application during a pandemic. Two articles [44,47] described uncontrolled pre–post studies that examined brief psychotherapeutic interventions (Brief School-based Cognitive Behavioral Intervention and StArT) for survivors of natural hazards (i.e., earthquakes and hurricanes). Both studies conducted with children and/or adolescents found that PTSD symptoms significantly decreased post-intervention.

Seven published articles or study protocols described brief psychotherapeutic interventions originally developed for survivors of other severe stressors, such as terrorism or traumatic events. Most of these interventions can also be applied to other types of stressors, such as disasters or pandemics. Overall, the results of five studies showed that the interventions EFT, exposure-based CBT, EMDR, MBSR, CBT-PD, and the Trauma Therapy Program were successful in reducing psychological distress and/or subclinical symptoms [49,52,56,57,60]. One RCT on the Preventive Resilience Training for Unaccompanied Refugee Minors reported no reduction in anxiety, PTSD, and depressive symptoms at a seven-week follow-up, although well-being significantly increased [53]. The reasons for the lack of effectiveness could be that the treatment dose was too low or the assessment of outcomes at only 7 weeks post-intervention might not be enough time to observe significant changes in symptoms. One study protocol described planning to examine the efficacy of SFBT on anxiety and depressive symptoms during the COVID-19 pandemic [63].

Overall, these findings suggest that there is the first evidence, especially on the efficacy of exposure-based CBT, EMDR, and EFT based on one RCT each. Additionally, there is a preliminary indication based on one uncontrolled pre–post study each that CBT-PD, MBSR, and Trauma Therapy Program may be effective in reducing psychological distress and/or subclinical symptoms.

### 5.4. Limitations

The interpretation of results synthesized in this systematic review should be interpreted considering the quality of the included studies. Thus, this systematic review is not without limitations. Several studies consisted of small sample sizes; more than half of the included studies considered fewer than 100 participants. Although a larger sample size would be desirable to achieve greater statistical power and generalizability in the results, there are challenges such as resource limitations and participant dropout making this difficult. In addition to 15 RCTs, 9 uncontrolled pre–post studies and 3 controlled pre–post studies were included in this review. Pre–post studies suffer from impaired internal validity and the associated limited interpretability of results. Furthermore, the long-term effectiveness of some interventions cannot be assessed because ten completed studies on these interventions did not report follow-up measurements [21,27,35,36,37,40,41,47,53,57]. One study lacked reporting on the length of the intervention [21]; ten studies did not report on the timepoint of intervention after stressor exposure [33,35,36,37,38,41,53,56,57,60], making it difficult to interpret the effectiveness of these interventions. Although we performed an extensive systematic review covering multiple established literature databases, we might have missed some additional studies describing effective interventions. We included six study protocols that described novel interventions for which evaluation was planned or ongoing and for which no evidence on their efficacy was available yet. Another drawback might arise from the nature of a systematic review to summarize and synthesize study results descriptively. Thus, it was not possible to directly compare the different interventions in terms of their efficacy, as could be performed in a meta-analysis. A strength of this review is the comprehensive reporting and discussion of the study’s findings; however, no quality assessment of the included studies was undertaken.

This systematic review extends previous research since, to our knowledge, no systematic review of studies describing or evaluating brief interventions aiming at reducing psychological distress and/or subclinical symptoms has been conducted before. We considered different types of stressors (i.e., natural hazards and man-made disasters, pandemics, and other severe stressors) as well as different types of interventions (i.e., self-help, psychosocial support, and psychotherapeutic) to provide a broad overview of brief interventions for reducing distress and/or subclinical symptoms. Therefore, some of the interventions and target groups might not be directly comparable.

## 6. Conclusions

This systematic review identified novel brief self-help programs, psychosocial support programs, or brief psychotherapeutic interventions that addressed distress and/or subclinical symptoms in survivors of disasters, pandemics, and other severe stressors. A few interventions showed the first evidence of being effective in reducing psychological distress and/or subclinical PTSD symptoms. Effective interventions mostly covered psychosocial support programs and brief psychotherapeutic interventions that focused on disasters or other severe stressors. Interventions that focused on the COVID-19 pandemic mainly involved self-help programs that showed limited evidence of effectiveness. Future research should further investigate the effectiveness of psychosocial support interventions and brief psychotherapeutic interventions for COVID-19 survivors.

## Figures and Tables

**Table 1:** Characteristics of the included published articles/protocols on brief interventions (*n* = 27).

Characteristic	*n*	%
Continent/Country		
Africa	**1**	**3.70**
Kenya	1	3.70
Asia	**9**	**33.33**
China	4	14.81
Nepal	2	7.41
India	1	3.70
Japan	1	3.70
Pakistan	1	3.70
Oceania	**3**	**11.11**
Australia	2	7.41
Polynesia	1	3.70
Europe	**5**	**18.52**
Germany	1	3.70
Austria	1	3.70
France	1	3.70
Netherlands	1	3.70
United Kingdom (UK)	1	3.70
America	**9**	**33.33**
United States (US)	7	25.93
Canada	1	3.70
Haiti	1	3.70
Sample size of studies/protocols		
1–50	6	22.22
51–100 ^a^	8	29.63
101–200 ^a^	3	11.11
201–400 ^b^	3	11.11
401–600	3	11.11
601–1000	2	7.41
>1000 ^b^	2	7.41
Study design of studies/protocols		
Randomized controlled trial	15	55.56
Controlled pre–post study	3	11.11
Uncontrolled pre–post study	9	33.33
Type of stressor		
Natural hazard	11	40.74
Pandemics	8	29.63
Another severe stressor	5	18.52
Man-made disaster	3	11.11
Type of intervention		
Self-help	6	22.22
Brief psychotherapeutic treatment	9	33.33
Psychosocial support	12	44.44

Note. ^a^ *n* = 2 included study protocols. ^b^ *n* = 1 included study protocol.

## Data Availability

Available upon reasonable request to the corresponding author.

## Appendix A

**Table A1:** Characteristics of brief interventions described in the included articles/protocols (*n* = 27).

Study	Intervention	Design	Sample Size	Type of Stressor	Aim of Intervention	Delivery Format	Target Population	No. of Sessions	Results
Self-help programs									
Liu et al. (2021) [20]	Computerized cognitive behavioral therapy (cCBT) [20]	RCT	*N* = 252	COVID-19 pandemic	Reduction in psychological distress.	Online	Adults	A total of 7 for at least 10 min.	cCBT was effective in reducing depression and anxiety at 1-month follow-up.
Zhou et al. (2020) [21]	Individualized Short-term Training Program [21]	Uncontrolled pre–post study	*N* = 71	COVID-19 pandemic	Reduction in depression and anxiety symptoms.	Hybrid	Adults	Not reported	The Individualized Short-term Training Program was effective in reducing anxiety but not depression at post-training.
Alavi et al. (2020) [22]	Online psychotherapy tool (OPTT) [22]	Controlled pre–post study	*N* = 80	COVID-19 pandemic	Reduction in mental health symptoms	Online	Adults	9	Not applicable (study protocol).
Weiner et al. (2020) [23]	“My health too” [23]	RCT	*N* = 120	COVID-19 pandemic	Reduction in distress. Prevention of long-term distress consequences.	Online	Adults	7	Not applicable (study protocol).
Gilmore et al. (2021) [25]	Bounce Back Now (BBN) [24]	Controlled pre–post study	*N* = 979	Tornadoes	Improvement in post-disaster mental health. Reduction in behavioral problems.	Online	Adolescents and parents	4	BBN was effective in reducing PTSD symptoms at 4- and 12-months follow-up.
Price et al. (2013) [27]	Disaster Recovery Web (DRW) Project [26]	Uncontrolled pre–post study	*N* = 1249	Hurricane	Reduction in symptoms of PTSD, depression, and anxiety.	Online	Adults	4	The DRW Project was not effective in reducing depressive symptoms or PTSD symptoms at 4-month post-intervention.
**Psychosocial support programs**									
Tang et al. (2021) [28]	Grief Counseling for Adults [28]	RCT	*N* = 160	Loss and grief during COVID-19	Promotion of life adaption after loss	Online	Adults	A total of 8–10 for 1 h each	Not applicable (study protocol)
Devassy et al. (2021) [32]	Resiliency Engagement and Care in Health (REaCH) [32]	cRCT	*N* = 1440	COVID-19 pandemic	Reduction in mental health symptoms. Increase in social support	Phone-based	Adults	Four	Not applicable (study protocol)
James et al. (2020) [33]	Mental Health Integrated Disaster Preparedness (MHIDP) Intervention [33]	RCT	*N* = 480	Earthquakes and floods	Improvement in disaster preparedness. Reduction in mental health symptoms. Promotion of community cohesion	Face-to-face	Adults	A total of 3 for 6 h each	The MHIDP Intervention was effective in reducing depressive, anxiety, and PTSD symptoms at 3–4-month and 7–8-month follow-up.
Sangraula et al. (2020) [35]	Problem Management Plus (PM+) [34]	cRCT	*N* = 121	Earthquake	Reduction in psychological distress	Face-to-face	Adults	A total of 5 for 2.5–3 h each	Group PM+ was effective in reducing depressive and PTSD symptoms, as well as psychological distress at 8 weeks post-intervention.
Jordans et al. (2021) [36]	Problem Management Plus (PM+) [34]	cRCT	*N* = 611	Disaster-prone communities (i.e., landslides or flooding)	Reduction in psychological distress	Face-to-face	Adults	A total of 5 for 2.5 h each	Group PM+ was effective in reducing depressive symptoms and psychological distress, but not PTSD symptoms, at 3-months post-treatment.
Rahman et al. (2016) [37]	Problem Management Plus (PM+) [34]	RCT	*N* = 60	Conflict-affected area	Reduction in psychological distress	Face-to-face	Adults	Five	PM+ was effective in reducing PTSD symptoms, but not psychological distress, at post-intervention.
Bryant et al. (2017) [38]	Problem Management Plus (PM+) [34]	RCT	*N* = 421	Physical and sexual abuse	Reduction in psychological distress	Face-to-face	Adults	A total of 5 for 90 min each	PM+ was effective in reducing psychological distress and PTSD symptoms at 3-months follow-up.
Keyan et al. (2021) [39]	Problem Management Plus (PM+) [34]	RCT	*N* = 240	COVID-19 pandemic	Reduction in psychological distress	Online	Adults	A total of 6 for 60 min each	Not applicable (study protocol)
O’Donnell et al. (2020) [6]	Skills fOr Life Adjustment and Resilience (SOLAR) [6]	Uncon-trolled pre–post study	*N* = 15	Bushfire	Reduction in post-disaster distress	Face-to-face	Adults	A total of 4 for 50 min each The first for 80 min.	SOLAR was effective in reducing psychological distress and post-traumatic stress symptoms at post-treatment.
Gibson et al. (2021) [40]	Skills fOr Life Adjustment and Resilience (SOLAR) [6]	Controlled pre–post study	*N* = 99	Tropical Cyclone	Reduction in post-disaster distress	Face-to-face	Adults	Five	SOLAR was effective in reducing psychological distress and PTSD symptoms at post-intervention.
Lotzin, Hinrichsen, Kenntemich, Freyberg, Lau, & O’Donnell (2021) [41]	Skills fOr Life Adjustment and Resilience (SOLAR) [6]	RCT	*N* = 30	Traumatic events	Reduction in post-disaster distress	Face-to-face	Adults	Five	SOLAR was effective in reducing psychological distress, but not PTSD symptoms, at post-intervention.
Ramirez et al. (2013) [43]	Listen Protect Connect (LPC) [42]	Uncontrolled pre–post study	*N* = 20	Flood	Provision of psychological support Reduction in distress	Face-to-face	Children	A total of 1 for 25 min on average	LPC marginally reduced PTSD symptoms at 8 weeks post-intervention and significantly reduced depressive symptoms at 4 weeks post-intervention.
**Brief psychotherapeutic programs**									
Ito et al. (2016) [44]	Brief School-Based Cognitive Behavioral Intervention [44]	Uncontrolled pre–post study	*N* = 22	Earthquake	Reduction in PTSD and depressive symptoms	Face-to-face	Adolescents	A total of 1 for 90 min	The Brief School-Based Cognitive Behavioral Intervention was effective in reducing PTSD symptoms, but not depressive symptoms, at post-intervention and 4-month follow-up.
Taylor & Weems (2011) [47]	Strength after Trauma, (StArT) [46]	Uncontrolled pre–post study	*N* = 6	Hurricane	Reduction in PTSD symptoms	Face-to-face	Children and adolescents	A total of 10 for 1 h each	StArT was effective in reducing PTSD symptoms, but not anxiety symptoms, at post-intervention.
Hamblen et al. (2017) [49]	Cognitive Behavioral Therapy for Post-disaster Distress (CBT-PD) [48]	Uncontrolled pre–post study	*N* = 342	Hurricane	Reduction in post-disaster distress	Face-to-face	Adults	A total of 10	CBT-PD was effective in reducing psychological distress over the course of the intervention until 5-month follow-up.
de Roos et al. (2011) [52]	Exposure-based Cognitive Behavioral Therapy, CBT [50] and Eye Movement Desensitization and Reprocessing (EMDR) [51]	RCT	*N* = 52	Explosion	Reduction in trauma-related distress and PTSD symptoms	Face-to-face	Children and adolescents	Up to 4 for 60 min.	Exposure-based CBT was effective in reducing PTSD, anxiety, and depressive symptoms at post-intervention. EMDR was effective in reducing PTSD, anxiety, and depressive symptoms at post-intervention.
Scheiber et al. (2019) [53]	Preventive Resilience Training for Unaccompanied Refugee Minors [53]	RCT	*N* = 55	Flight	Reduction in symptoms of PTSD, depression, and anxiety	Face-to-face	Adolescents	A total of 6 for 90 min each	The Preventive Resilience Training was not effective in reducing anxiety, PTSD, and depressive symptoms at 7 weeks post-intervention.
Gallegos et al. (2015) [56]	Mindfulness-Based Stress Reduction (MBSR) [54]	Uncontrolled pre–post study	*N* = 50	Childhood trauma	Improvement in mindfulness	Face-to-face	Adults	A total of 8 for 120 min each and 1 retreat (4 h)	MBSR was effective in reducing perceived distress, depressive symptoms, anxiety, and PTSD symptoms at post-intervention and at 1-month follow-up.
Tehrani (2019) [57]	Trauma Therapy Program [57]	Uncontrolled pre–post study	*N* = 429	Traumatic events in emergency service	Reduction in work-related psychological symptoms	Face-to-face	Adults	A total of 6 for 90 min each	The Trauma Therapy Program was effective in reducing anxiety, depression, and PTSD symptoms at post-treatment.
Church et al. (2013) [60]	Emotional Freedom Techniques (EFT) [59]	RCT	*N* = 55	War	Reduction in psychological distress and symptoms of depression, anxiety, and PTSD	Face-to-face	Adults	A total of 6 for 60 min each	EFT was effective in reducing PTSD, anxiety, and depression at post-intervention until 3-month and 6-month follow-up.
Chen (2020) [63]	Solution-Focused Brief Therapy (SFBT) [61]	RCT	*N* = 76	COVID-19 pandemic	Reduction n mental health symptoms	Online	Adolescents	A total of 2–4	Not applicable (study protocol)

**Table A2:** Detailed overview of intervention characteristics and results of the included studies/protocols (*n* = 27).

Study	Intervention Name and Description	Study Design	Timepoint of Intervention after Stressor Exposure	No. of Sessions	Type of Stressor	Target Population	Delivery Format	Assessed Outcomes	Results
Self-help programs									
Liu et al. (2021) [20]	Computerized Cognitive Behavioral Therapy (cCBT) [20]: Online self-help cCBT targeting patients with COVID-19 A total of three modules: cognitive training, cognitive consolidation, and behavioral therapy Introduction to the program by a therapist	RCT (Intervention + TAU vs. TAU)	During COVID-19	A total of 7 at least for 10 min.	COVID-19	*N* = 252 adults with COVID-19 Mild to moderate depressive or anxiety symptoms (HAMD, score ≥ 7; HAMA score ≥7) Age (range: 18–75 years) cCBT + TAU (*n* = 126): 56% female TAU (*n* = 126): 46% female	Online	Primary outcomes: Symptoms of depression: Hamilton Depression Rating Scale, HAMD [64] Symptoms of Anxiety: Hamilton Anxiety Scale, HAMA [65] Secondary outcomes: Depression: Self-Rating Depression Scale, SDS [66] Anxiety: Self-Rating Anxiety Scale, SAS [67] Insomnia: Athens Insomnia Scale, AIS [68]	Significantly decreased score of the cCBT + TAU group on the HAMD, HAMA, SDS, SAS, and AIS at post-intervention compared to the TAU group (all *p* < 0.001) HAMD cCBT + TAU: T_0_: *M* = 15.28; Post: *M* = 7.86; 1-month FU: *M* = 6.68; TAU: T_0_: *M* = 15.70; Post: *M* = 15.46; 1-month FU: *M* = 15.26 HAMA cCBT + TAU: T_0_: *M* = 14.26; Post: *M* = 7.38; 1-month FU: *M* = 6.10; TAU: T_0_: M = 13.88; Post: *M* = 13.24; 1-month FU: *M* = 13.20 SDS cCBT + TAU: T_0_: *M* = 46.10; Post: *M* = 32.56; 1-month FU: *M* = 31.14; TAU: T_0_: M = 45.22; Post: *M* = 45.56; 1-month FU: *M* = 44.70 SAS cCBT + TAU: T_0_: *M* = 44.30; Post: *M* = 29.66; 1-month FU: *M* = 29.12; TAU: T_0_: *M* = 45.56; Post: *M* = 45.52; 1-month FU: *M* = 44.92 AIS cCBT + TAU: T_0_: *M* = 8.58; Post: *M* = 6.98; 1-month FU: *M* = 6.88 TAU: T_0_: *M* = 8.20; Post: *M* = 8.00; 1-month FU: *M* = 7.82
Zhou et al. (2020) [21]	Individualized Short-term Training Program [21]: Short-term online and in-person emergency training Covers knowledge about diagnosing COVID-19, handling and safety precautions with infected patients, and psychological support including mindfulness-based stress reduction Delivered by psychologists	Uncontrolled pre–post study	During COVID-19	Not reported	COVID-19	*N* = 71 female nursing staff working in the emergency isolation department Age (*M* = 31.31; *SD* = 4.85)	Hybrid (online and in-person)	Primary outcomes: Symptoms of anxiety: Self-rating Anxiety Scale, SAS [67] Symptoms of depression: Self-rating Depression Scale, SDS [66]	Comparison of in-person and online + in-person training: Better evaluation of theoretical training (*p* = 0.042) and drill training (*p* = 0.002) using online + in-person training method. No difference in evaluation of operation training between the two methods (*p* = 0.081). Lower SAS score post-training (*p* = 0.019) Mean difference in SAS scores between T_0_ and post-training: *M* = -3.06 No significant reduction in SDS score (*p* = 0.31) Mean difference in SDS scores between T_0_ and post-training: *M* = -1.99
Alavi et al. (2020) [22]	Online Psychotherapy Tool (OPTT) [22]: CBT-program via an online platform Combination of CBT, mindfulness therapy, and problem-based therapy Weekly self-guided modules and written feedback from trained therapists	Controlled pre–post study (Intervention vs. TAU)	During COVID-19	Nine	COVID-19	*N* = 80 adults aged 18–65 years with a primary diagnosis of GAD or MDD	Online	Primary outcomes: Anxiety: Generalized Anxiety Disorder-7, GAD-7 [69] Depressive symptoms: Patient Health Questionnaire-9 Item, PHQ-9 [70] Resilience: Resilience Scale-14 Item Questionnaire, RS-14 [71] Quality of life: Quality of Life and Enjoyment Questionnaire, Q-LES-Q [72]	Not applicable (study protocol)
Weiner et al. (2020) [23]	“My health too” [23]: Online self-help CBT program Seven asynchronous video sessions: psychoeducation, functional behavioral and cognitive coping strategies, mindfulness, mindfulness/acceptance, promoting action toward values, addressing barriers and motivation, and self-compassion Optional psychotherapeutic support	RCT (Intervention vs. Control)	During COVID-19	Seven	COVID-19	*N* = 120 healthcare workers with stress levels > 16 on the Perceived Stress Scale (PSS-10)	Online	Primary outcome: Stress: Perceived Stress Scale, PSS-10 [73] Secondary outcomes: Depressive symptoms: Patient Health Questionnaire, PHQ-2 [74] PTSD symptoms: Short Form Post-traumatic Stress Disorder Checklist 5, SF-PCL-5 [75] Resilience: Connor-Davidson Resilience Scale, CD-RISC2 [76] Insomnia: Insomnia Severity Index, ISI [77] Rumination: Affective Rumination Questionnaire, ARQ [78] Credibility of treatment: Credibility and Expectancy Questionnaire, CEQ [79] Satisfaction: Client Satisfaction Questionnaire, CSQ-8 [80]	Not applicable (study protocol)
Gilmore et al. (2021) [25]	Bounce Back Now (BBN) [24]: Web-based self-help intervention for disaster-affected adolescents and parents Four modules: PTSD, depression (mood), smoking, and alcohol use BBN+ ASH (Bounce Back Now plus a seven-module adult self-help (ASH) intervention)	Controlled pre–post study (Intervention vs. Control)	*M* = 8.8 (*SD* = 2.6) months after tornado	Four	Tornadoes in Joplin, Missouri, and several areas of Alabama, 2011	*N* = 979 adolescents 52.9% female Age (*M* = 14.3; *SD* = 1.7)	Online	Primary outcome: PTSD symptoms: National Survey of Adolescents (NSA) PTSD module [81]	BBN > Control Significant decline in PTSD symptoms in BBN condition over time (*b* = −0.02, *p* = 0.04, OR = 0.98) with adolescents who had caregivers who were concerned for loved ones during the disaster No significant decline in PTSD symptoms in control condition over time (*b* = 0.02, *p* = 0.26, OR = 1.02) PTSD symptoms BBN: T_0_: *M* = 1.35 (*SD* = 2.43); 4-Month FU: *M* = 1.25 (*SD* = 2.59); 12-Month FU *M* = 1.12 (*SD* = 2.54) PTSD symptoms control: T_0_: *M* = 1.45 (*SD* = 2.45); 4-Month FU *M* = 1.18 (*SD* = 2.47); 12-Month FU *M* = 1.26 (*SD* = 2.15)
Price et al. (2013) [27]	Disaster Recovery Web (DRW) Project [26]: Web-based self-help intervention Four modules: post-traumatic stress, depressed mood, generalized anxiety, and panic	Uncontrolled pre–post study	1 year after the stressor	Four	Hurricane Ike in Texas, 2008	*N* = 1249 adults who survived Hurricane Ike with symptoms of PSTD, depression, and anxiety. Equally distributed across genders Age (*M* = 46; *SD* = 17)	Online	Primary outcomes: Symptoms of PTSD: The PTSD Checklist-Civilian version, PCL-C [82] Depressive Symptoms: Center for Epidemiologic Studies-Depressed Mood Scale-10, CES-D [83]	No significant reduction in PTSD symptoms and depressive symptoms between T_0_ and 4-month post-intervention
**Psychosocial support programs**									
Tang et al. (2021) [28]	Grief Counseling [28]: Online Grief Counseling based on CBT Topics: understanding and managing grief reactions, managing painful emotions, learning to care for yourself, increasing contact with others, coping with difficult days, and adapting to a new life Delivered by psychologists, social workers, or trained counselors	RCT (Intervention vs. Wait list control)	After stressor at any timepoint	A total of 8–10 for 1 h each	COVID-19	*N* = 160 participants aged > 18 who have lost their first-degree relatives during COVID-19	Online	Primary outcomes: Symptoms of PTSD: PTSD Checklist for DSM-5, PCL-5 [84] Post-traumatic Growth Inventory, PTGI [85] Depressive, anxiety, and stress symptoms: Depression Anxiety and Stress Scale, DASS-21 [86] Grief symptoms: Prolonged Grief Questionnaire, PG-13 [87] Secondary outcomes: Suicidal intention: Scale for Suicidal Intention, SSI [88] Maladaptive cognitions: Typical Beliefs Questionnaire, TBQ [89] Avoidance behavior: Grief-related Avoidance Questionnaire, GRAQ [90] Functioning in relationships: The Work and Social Adjustment Scale, WSAS [91]	Not applicable (study protocol)
Devassy et al. (2021) [32]	Resiliency Engagement and Care in Health (REaCH) [32]: Telephonic befriending psychosocial intervention Four phone calls for 30 min-1 h consisting of proactive engagement and crisis intervention, problem-solving-oriented support therapy and assertive linkage with community resources Delivered by lay workers and non-health professionals	cRCT (Intervention vs. Control)	During COVID-19	Four	COVID-19	*N* = 1440 adults aged 18–35 years from economically disadvantaged and vulnerable sections of society	Phone-based	Primary outcomes: Mental well-being: World Health Organization-Five Well-Being Index, WHO-5 [92] Depressive symptoms: Patient Health Questionnaire, PHQ-9 [70] Perceived social support: Multidimensional Scale of Perceived Social Support, MSPSS-12 [93]	Not applicable (study protocol)
James et al. (2020) [33]	Mental Health Integrated Disaster Preparedness (MHIDP) Intervention [33]: Community-based mental health intervention Topics: establishing safety and practicing coping skills targeting disaster-related distress, providing space for sharing personal experiences, and giving hands-on training in disaster preparedness Delivered by lay mental health workers	RCT (Intervention vs. wait list control)	Not reported	A total of 3 for 6 h each	Earthquakes or floods in Haiti	*N* = 480 adults drawn from disaster-affected communities 49.8% female Age (*M* = 37; *SD* = 13.6)	Face-to-face	Primary outcomes: Disaster preparedness: Twenty-item disaster preparedness checklist [33] Symptoms of PTSD: Modified PTSD Symptom Scale, MPSS [94] Symptoms of depression: Zanmi Lasante Depression Symptom Inventory, ZLDSI [95] Symptoms of anxiety: Beck Anxiety Inventory, BAI [96] Functional impairment [96] Social cohesion [97]	Highly significant unstandardized regression coefficients (*p* < 0.001) to indicate the change in scale values in the intervention group relative to control from T_0_ to 3–4-month FU Disaster preparedness: 4.18 (*d* = 0.75) Depression: −0.35 (*d* = −0.47) PTSD: −0.46 (*d* = −0.49) Anxiety: −0.27 (*d* = −0.41) Significant unstandardized regression coefficients (*p* < 0.05) from T_0_ to 3–4-month FU in functional impairment: −0.35 (*d* = 0.29) which disappeared at 7–8-month FU Highly significant unstandardized regression coefficients (*p* < 0.001) from 3–4-month FU to 7–8-month FU in disaster preparedness: 2.90 (*d* = 0.52)
Keyan et al. (2021) [39]	Problem Management Plus (PM+) [34]: Brief psychosocial intervention Adaption to be delivered in a group setting (Group PM+) Four strategies: managing stress, managing problems, behavioral activation, strengthening social support Delivered by trained nonspecialist lay providers	RCT (Intervention vs. ETAU)	During COVID-19	A total of 6 for 60 min each	COVID-19	*N* = 240 adults with a score ≥ 3 on the GHQ-12 [98]	Online	Primary outcome: Anxiety and depressive symptoms: Hospital Anxiety and Depression Scales, HADS [99] Secondary outcomes: Generalized Anxiety: Generalized Anxiety Disorder Scale, GAD-7 [69] Sleep Impairment: Insomnia Severity Index, ISI [100] Mood: Positive and Negative Affect Schedule, PANAS [101] Anhedonia: Pleasure Scale [102] COVID stress: COVID Stress Scales, CSS [103]	Not applicable (study protocol)
Sangraula et al. (2020) [35]	Problem Management Plus (PM+) [34]: Brief psychosocial intervention Adaption to be delivered in a group setting (Group PM+) Four strategies: managing stress, managing problems, behavioral activation, strengthening social support Delivered by trained nonspecialist lay providers	cRCT (Intervention vs. EUC)	Not reported	A total of 5 for 2.5–3 h each	Earthquake-affected region of rural Nepal	*N* = 121 participants 83% female Group PM+ (*n* = 61): Age (*M* = 46.7; *SD* = 14) EUC (*n* = 60); Age (*M* = 49.3; *SD* = 13.6)	Face-to-face	Primary outcome: Depressive symptoms: Primary Health Questionnaire, PHQ-9 [70] Secondary outcomes: Psychological distress: General Health Questionnaire, GHQ-12 [98]; Heart–mind screener [104] Daily functioning: WHO Disability Assessment Scale, WHODAS [105] PTSD symptoms: Post-traumatic stress disorder Check List, PCL-5 [84] Psychosocial problems: Psychosocial Mental Health Problems, PMHP [106] Social Support: Multidimensional Scale of Perceived Social Support, MSPSS [93] Coping strategies: Reducing Tension Checklist, RTC [107] Traumatic events: Traumatic Events Inventory, TEI [108] Personally identified problems: Psychological Outcome Profiles, PSYCHLOPS [109]	Feasibility and acceptability for nonspecialists to deliver Group PM+ PHQ-9: T_0_ PM(PM+; [34]): *M* = 9.7 (*SD* = 4.8); T_0_ EUC: *M* = 10.9 (*SD* = 4.3) Post PM(PM+; [34]): *M* = 6.2 (*SD* = 3.7); Post EUC: *M* = 9.3 (*SD* = 4.3) WHODAS: T_0_ PM(PM+; [34]): *M* = 21.5 (*SD* = 4.9); T_0_ EUC: *M* = 20.9 (*SD* = 4.2) Post PM(PM+; [34]): *M* = 12.1 (*SD* = 8.0); Post EUC: *M* = 15.7 (*SD* = 6.4) GHQ-12: T_0_ PM(PM+; [34]): *M* = 24.2 (*SD* = 4.8); T_0_ EUC: *M* = 21.4 (*SD* = 4.8) Post PM(PM+; [34]): *M* = 11.9 (*SD* = 6.6); Post EUC: *M* = 17.6 (*SD* = 6.0) PMH(PM+; [34]): T_0_ PM(PM+; [34]): *M* = 10.1 (*SD* = 3.3); T_0_ EUC: *M* = 11.2, (*SD* = 2.7) Post PM(PM+; [34]): *M* = 9.1, (*SD* = 3.0); Post EUC: *M* = 11.2, (*SD* = 2.9) PCL-5: T_0_ PM(PM+; [34]): *M* = 17.5 (*SD* = 7.2); T_0_ EUC: *M* = 21.8 (*SD* = 5.7) Post PM(PM+; [34]): *M* = 14.8 (*SD* = 8.1); Post EUC: *M* = 20.5 (*SD* = 5.6) RTC: T_0_ PM(PM+; [34]): *M* = 15.6 (*SD* = 4.8); T_0_ EUC: *M* = 10.2 (*SD* = 5.1) Post PM(PM+; [34]): *M* = 20.6 (*SD* = 5.8); Post EUC: *M* = 9.4 (*SD* = 4.2) MSPSS: T_0_ PM(PM+; [34]): *M* = 33.3 (*SD* = 8.0); T_0_ EUC: *M* = 29.6 (*SD* = 8.7) Post PM(PM+; [34]): *M* = 34.2 (*SD* = 7.0), Post EUC: *M* = 29.4 (*SD* = 8.7)
Jordans et al. (2021) [36]	Problem Management Plus (PM+) [34]: Brief psychosocial intervention Adaption to be delivered in a group setting (Group PM+) Four strategies: managing stress, managing problems, behavioral activation, strengthening social support Delivered by trained nonspecialist lay providers	cRCT (Intervention vs. EUC)	Not reported	A total of 5 for 2.5 h each	Disaster-prone regions in Nepal	*N* = 611 adults screened for psychological distress and functional impairment in 72 eligible wards 82.2% female Age (*M* = 44.8; *SD* = 14.4)	Face-to-face	Primary outcome: General psychological distress: General Health Questionnaire, GHQ-12 [98] Secondary outcomes: Depressive symptoms: Primary Health Questionnaire, PHQ-9 [70] Daily functioning: WHO Disability Assessment Scale, WHODAS [105] PTSD symptoms: Post-traumatic stress disorder Check List, PCL-5 [84] Perceived social support: Multi-dimensional Scale of Perceived Social Support, MSPSS [93] Somatic symptoms: Somatic Symptom Scale 8, SSS-8 [110] General psychological distress: Heart–mind screener (Community Informant Detection Tool, CIDT [111]	Group-PM+ > EUC#breakLower distress in the PM+ Group at both midline (SMD = −0.4 (95% CI: −0.5, 0.0.2); *p* < 0.001) and endline (SMD = −0.2 (95% CI: −0.4, −0.0); *p* = 0.014) compared to the control arm#breakLower depression symptoms of the Group-PM+ arm at endline (PHQ-9 mean difference = −1.0, 95% CI: −1.8, −0.1, *p* = 0.028)#breakGroup-PM+ arm had fewer “heart-mind” problems at endline (risk ratio = 0.8 (95% CI: 0.7, 1.0, *p* = 0.042))#breakGroup-PM+ = EUC#breakGroup-PM+ was not associated with lower functional impairment (WHODAS mean difference = 1.5, 95% CI: −3.4, 0.4, *p* = 0.118), PTSD symptoms (PCL mean difference = −1.0, 95% CI: −2.2, 0.1, *p* = 0.084), perceived social support (MSPSS mean difference = 1.0, 95% CI: 0.0.3, 2.3, *p* = 0.138), nor somatic symptoms (SSS mean difference = −1.0, 95% CI: −2.2, 0.2, *p* = 0.105)
Rahman et al. (2016) [37]	Problem Management Plus (PM+) [34]: Brief psychosocial intervention Adaption to be delivered in a group setting (Group PM+) Four strategies: managing stress, managing problems, behavioral activation, strengthening social support Delivered by trained nonspecialist lay providers	RCT (Intervention vs. ETAU)	Not reported	Five	Conflict-affected Peshawar in Pakistan	*N* = 60 participants with both marked distress and impairment	Face-to-face	Primary outcome: Psychological distress: General Health Questionnaire, GHQ-12 [98] Secondary outcomes: Daily functioning: WHO Disability Assessment Scale, WHODAS [105] PTSD symptoms: Post-traumatic stress disorder Check List, PCL-5 [84]	PM+ > ETAU The intervention arm showed improvement in functioning (mean WHODAS 2.0 scores reduced from 17.7 ± 9.2 to 6.6 ± 6.1 vs. 17.0 ± 10.5 to 11.3 ± 10.4 in controls) and in post-traumatic stress symptoms (mean PCL-5 scores reduced from 34.2 ± 20.1 to 9.8 ± 9.1 vs. 32.3 ± 17.1 to 19.5 ± 18.5 in controls) PM+ = ETAU No significant change in GHQ-12 scores
Bryant et al. (2017) [38]	Problem Management Plus (PM+) [34]: Brief psychosocial intervention Adaption to be delivered in a group setting (Group PM+) Four strategies: managing stress, managing problems, behavioral activation, strengthening social support Delivered by trained nonspecialist lay providers	RCT (Intervention vs. EUC)	Not reported	A total of 5 for 90 min each	Gender-based violence (GBV) in Kenya	*N* = 421 women Age (*M* = 35.56; *SD* = 13.39)	Face-to-face	Primary outcome: Psychological distress: General Health Questionnaire, GHQ-12 [98] Secondary outcomes: Daily functioning: WHO Disability Assessment Scale, WHODAS [105] PTSD symptoms: Post-traumatic stress disorder Check List, PCL-5 [84] Personally identified problems: Psychological Outcome Profiles, PSYCHLOPS [109] Stressful life events: Life Events Checklist, LEC [112]	PM+ > EUC Greater reduction in distress from baseline to 3 months (95% CI 1.86 ± 4.79, *p* = 0.001) in the PM+ Group For WHODAS the difference between PM+ and EUC in the change from baseline to 3-month follow-up was 1.96 (95% CI 0.21 ± 3.71, *p* = 0.03), for PCL it was 3.95 (95% CI 0.06 ± 7.83, *p* = 0.05), and for PSYCHLOPS it was 2.15 (95% CI 0.98 ± 3.32, *p* = 0.001), all in favor of PM+. Moderate effect sizes in favour of PM+ for GHQ-12 score (0.57, 95% CI 0.32 ± 0.83) and PSYCHLOPS (0.67, 95% CI 0.31 ± 1.03), and small effect sizes for WHODAS (0.26, 95% CI 0.02 ± 0.50) and PCL (0.21, 95% CI 0.00 ± 0.41). PM+ = EUC For the LEC the between-group difference at 3-month follow-up was 0.31 (95% CI 0.02 ± 1.23, *p* = 0.51), indicating no difference in exposure to stressful life events between the groups. There was a very small between-group effect size (0.03, 95% CI −0.23 to 0.15).
O’Donnell et al. (2020) [6]	Skills fOr Life Adjustment and Resilience program (SOLAR) [6]: Brief disaster-focused psychosocial intervention#breakSix modules: healthy living, managing strong emotions, getting back into life, coming to terms with the disaster, managing worry and rumination, maintaining healthy relationships#breakDelivered by trained coaches	Uncontrolled pre–post study	1 year after stressor exposure	A total of 4 for 50 min each. The first for 80 min	Australian Bushfires	*N* = 15 adults impacted by bushfires with subclinical anxiety, post-traumatic stress, or depression symptoms, and distress 53.3% female Age (*M* = 58.68, *SD* = 11.53)	Face-to-face	Primary outcomes: Psychological distress: Kessler Psychological Distress Scale, K10 [113] PTSD symptoms: PTSD Checklist for DSM-5, PCL-5 [84] Secondary outcomes: Single impairment item [6] Psychological Outcome Profiles instrument, PSYCHLOPS [109]	Pre–post quantitative analysis demonstrated reductions in psychological distress, post-traumatic stress symptoms, and impairment (*p* < 0.05) K10: T_0_: *M* = 18.40 (*SD* = 5.01); Post: *M* = 13.08 (*SD* = 2.36); 3-Month FU: *M* = 13.73 (*SD* = 2.81) PCL-5: T_0_: *M* = 17.87 (*SD* = 8.29); Post: *M* = 5.07 (*SD* = 5.65); 3-Month FU *M* = 6.93 (*SD* = 6.51) PSYCHLOPS: T_0_: *M* = 11.79 (*SD* = 4.39); Post: *M* = 5.25 (*SD* = 2.30); 3-Month FU *M* = 5.67 (*SD* = 2.84) Impairment: T_0_: *M* = 4.64 (*SD* = 1.95); Post: *M* = 1.07 (*SD* = 1.54); 3-Month FU *M* = 2.2 (*SD* = 2.27)
Gibson et al. (2021) [40]	Skills fOr Life Adjustment and Resilience program (SOLAR) [6]: Brief disaster-focused psychosocial intervention#breakSix modules: healthy living, managing strong emotions, getting back into life, coming to terms with the disaster, managing worry and rumination, maintaining healthy relationships#breakDelivered by trained coaches	Controlled pre–post pilot study (Intervention vs. UC)	3 years and 7 months after stressor exposure	Five	Tropical Cyclone Pam, 2015	*N* = 99 residents of Tuvalu exposed to Tropical Cyclone Pam across Nui (*n* = 49) 76% female Age (*M* = 34.12; range: 18–71) and Funafuti (*n* = 50) 57% female Age (*M* = 50.02; range: 20–74)	Face-to-face	Primary outcome: Psychological distress: Hopkins symptom checklist-25 (HSCL-25) Tuvalu, HSCL-25 [114] Secondary outcomes: PTSD symptoms: PTSD Checklist for DSM-5, PCL-5) [84] Impairment: Tuvalu impairment checklist, TIC [114] Participant-identified difficulties: Psychological outcomes profiles, PSYCHLOPS [109]	Acceptability: High degree of session attendance of 4 sessions (*SD* = 1.25) on average; program was found to be useful and/or important; participants would recommend the program Feasibility: Pre–Post training analyses: Significant improvements in coaches’ knowledge of program content, *t*(10) = 4.36, *p* = 0.001, d_RM_ = 1.76, 95% CI: [0.41, 3.11]; their ability to apply that knowledge in response to example vignettes, *t*(10) = 19.10, *p* < 0.001, d_RM_ = 6.83, 95% CI: [3.15, 10.51]; and their confidence delivering the program, *t*(9) = 2.98, *p* = 0.015, d_RM_ = 1.26, 95% CI: [0.07, 2.45] Efficacy: Distress: SOLAR > UC Mean difference of 0.520 [95% CI: 0.646, 0.395], with the intervention group adjusted mean statistically significantly lower than that of the control group (Glass’ delta = 1.106 [0.839, 1.373]) PTSD symptoms: SOLAR > UC Large significant difference between groups (Glass’ delta = 1.575 [1.341, 1.810]), with significantly greater declines in PTSD symptoms in the intervention group Impairment: SOLAR > UC Greater reductions in impairment in the intervention group (Glass’ delta = 1.316 [1.117, 1.516])
Lotzin, Hinrichsen, Kenntemich, Freyberg, Lau, & O’Donnell (2021) [41]	Skills fOr Life Adjustment and Resilience program (SOLAR) [6]: Brief disaster-focused psychosocial intervention#breakSix modules: healthy living, managing strong emotions, getting back into life, coming to terms with the disaster, managing worry and rumination, maintaining healthy relationships#breakDelivered by trained coaches	RCT (Intervention vs. Wait list control)	Not reported	Five	Traumatic events	*N* = 30 German trauma survivors with subclinical symptoms of depression, anxiety, or post-traumatic stress disorder or functional impairment Age (*M* = 42) SOLAR group program (*n* = 15): 73.3% female Wait list control group (*n* = 15): 53.3% female	Face-to-face	Primary outcome: Feasibility: Client Satisfaction Questionnaire, CSQ-8 [115,116] Secondary outcomes: Psychological distress: Kessler Psychological Distress Scale-10, K10 [113] Symptoms of insomnia: Insomnia Severity Index, ISI [100] PTSD symptoms: PTSD Checklist for DSM–5, PCL-5 [84] Patient-centered outcomes: Psychological Outcome Profiles Scale, PSYCHLOPS [109] Quality of life: Assessment of Quality of Life-6D, AQoL-6D [117] Perceived social support: Interpersonal Support Evaluation List-12, ISEL-12 [118]	Feasibility: Among the 14 participants that started the SOLAR program, 13 (92.9%) completed 4 out of 5 sessions, 3 (10.0%) of the 30 randomized participants dropped out of the study, on average, participants were “very satisfied” (*M* = 3.85, *SD* = 0.44) with the program SOLAR > Wait list control Distress decreased in the intervention group but remained in the moderate/severe range in the control group (*d* = 0.195). Symptoms of insomnia decreased in the intervention group and marginally decreased in the control group (*d* = 0.596) Large effect sizes (*d* = 1.667) for patient-cenered outcomes: Severity of the problem causing the most distress declined in the intervention group but not in the control group Greater reduction in impairment in the intervention group relative to the control group (*d* = 0.362) Intervention group showed a greater improvement in quality of life including mental health (*d* = 0.642), relationships (*d* = 0.541), and problem coping (*d* = 0.548) Perceived social support increased more greatly in the intervention group relative to the control group (*d* = 0.560) SOLAR = Wait list control PTSD symptoms did not more greatly decrease in the intervention group relative to the control group (*d* = 0.032)
Ramirez et al. (2013) [43]	Listen Protect Connect (LPC) [42]: School-based crisis response strategy of PFA Five steps: Listen, Protect, Connect, Model, and Teach Delivered by school staff	Uncontrolled pre–post study	10 months after the stressor	A total of 1 for 25 min on average	Great Flood of Iowa, 2008	*N* = 20 children with personal trauma or expressed distress 20% female Age (range: 12–17 years)	Face-to-face	Primary outcomes: PTSD symptoms: Child PTSD Symptom Scale [119] Depressive symptoms: Center for Epidemiologic Studies Depression Scale, CES-D [83] Secondary outcomes: Social support: Multidimensional Scale of Perceived Support, MSPSS [93] School connectedness: Healthy Kids Resilience Measure of School Connectedness [120]	Marginally significant decrease in PTSD symptoms over time (*p* = 0.09) 3.7 points from T_0_ to the 8-week FU Significant decrease in depressive symptoms 2 weeks (adjusted *M* = 14.3; *p* < 0.01) and 4 weeks (adjusted *M* = 13.2; *p* < 0.01) after intervention and slightly increase 8 weeks (adjusted *M* = 15.2; *p* < 0.01) after intervention Social support increased from T_0_ to the 2-week FU (adjusted *M* = 3.9; *p* = 0.08), and increased significantly from T_0_ through 8-weeks (adjusted *M* = 4.0; *p* < 0.01) School connectedness was higher at 2- (*M* = 63.8; *p* = 0.06) and 4-weeks (*M* = 68.9, *p* < 0.01) than at T_0_ (*M* = 58.6), but this relationship diminished by 8-weeks
**Brief psychotherapeutic programs**									
Ito et al. (2016) [44]	Brief School-Based Cognitive Behavioral Intervention [44]: School-based cognitive behavioral intervention Four steps: identification of problems, psychoeducation, decreasing negative appraisal, and practice of relaxation breathing Delivered by clinical psychologists trained in CBT	Uncontrolled pre–post study	3 years after the stressor	A total of 1 90 min session	Great East Japan Earthquake, 2011	*N* = 22 adolescents with severe post-traumatic stress symptoms 15 female, 7 male Age (*M* = 15.4; *SD* = 0.5)	Face-to-face	Primary outcome: PTSD symptoms: Impact of Event Scale-Revised, IES-R [121] Secondary outcome: Depressive symptoms: Center for Epidemiologic Studies Depression Scale, CES-D [83]	Significant improvements in all post-traumatic stress symptoms at postintervention (*d* = 0.81, *p* = 0.01). IES-R Total: T_0_: *M* = 35.39 (*SD* = 10.19); Post: *M* = 24.95 (*SD* = 15.19) Effects were maintained throughout the 4-month FU period (*d* = 1.10, *p* < 0.001) IES-R Total: 4-month FU *M* = 19.32 (*SD* = 17.83) No significant reduction in depressive symptoms
Taylor & Weems [47]	Strength after Trauma (StArT) [46]: Manual-based hurricane trauma-focused CBT intervention Five modules: psychoeducation, cognitive restructuring, exposure, problem solving, and relapse prevention Delivered by a person experienced in psychotherapeutic treatment of adolescents	Uncontrolled pre–post study	4 years after the stressor	A total of 10 for 1 h each	Hurricane Katrina, 2005	*N* = 6 children exposed to Hurricane Katrina and/or its aftermath who met diagnostic criteria for PTSD 4 female, 2 male Age (range: 8–13 years)	Face-to-face	Primary outcomes: PTSD symptoms: Reaction Index for Children, PTSD-RI [122] Diagnostic Interview Schedule for Children-Predictive Scales, DISC-PS [123] Secondary outcomes: Negative cognitions: Children’s Negative Cognitive Error Questionnaire, CNCEQ [124] Anxiety sensitivity: Administration of the Childhood Anxiety Sensitivity Index, CASI [125] Control beliefs: Short form Anxiety Control Questionnaire for Children, ACQ-C [126]	Significant decline in PTSD symptoms (*d* = 2.00; *p* < 0.05) between pre- and posttreatment PTSD-RI: T_0_: *M* = 45.0 (*SD* = 11.6); Post: *M* = 15.0 (*SD* = 17.5) Significant decline in cognitive errors between pre- and post-treatment (*d* = 0.826; *p* < 0.05) CNCEQ: T_0_ *M* = 47.0 (SD = 25.1); Post *M* = 31.7 (SD = 7.39) No statistically significant differences in CASI, ACQ-C, and DISC-PS total and anxiety scores from T_0_ to Post
Hamblen et al. (2017) [49]	Cognitive behavioral therapy for post-disaster distress (CBT-PD) [48]: CBT intervention for post-disaster distress Three main sections: psychoeducation, coping skills, and cognitive restructuring#breakDelivered by trained therapists	Uncontrolled pre–post study	10–15 months or 21–26#breakmonths after the stressor	A total of 10	Hurricane Sandy, 2012	*N* = 342 adults with disaster-related symptoms exposed to Hurricane Sandy 80% female Age (*M* = 57; *SD* = 13)	Face-to-face	Primary outcome: PTSD symptoms: Short Post-Traumatic Stress Disorder Rating Interview–Expanded, Sprint-E [127]	2 × 3 × 4 (Severity × Timing × Session) mixed ANOVA: Large improvements in reduction in distress symptoms between pretreatment and intermediate (*M* diff = 6.47, *d* = 0.70) treatments and between intermediate and posttreatment (*M* diff = 6.90, *d* = 0.71) No effect of timing, but severity had a strong main effect on distress: Less severe group improved between referral and pretreatment (*M* diff = 2.96, *d* = 0.33), more severe group worsened over that time (*M* diff = 2.28, *d* = 0.31); more severe group improved between pretreatment and intermediate treatment (*M* diff = 9.42, *d* = 1.16, compared with *M* diff = 0.78, *d* = 0.09, in the less severe group). Improvements between intermediate treatment and posttreatment (for severe group, *M* diff = 6.96, *d* = 0.68; for moderate group, *M* diff = 6.79, *d* = 0.79) in both groups 5-Month FU: Slightly decrease in Sprint-E total scores in the moderate distress group (*M* diff = 1.30, *d* = 0.14), but increased slightly in the severe distress group (*M* diff = 2.64, *d* = 0.24)
de Roos et al. (2011) [52]	Exposure-based Cognitive Behavioral Therapy (CBT) [50]: CBT-based trauma treatment Elements: psychoeducation, repeated exposure to the trauma memory, cognitive restructuring, exploring, and correcting undesired or unhelpful coping behavior, and relapse prevention Eye Movement Desensitization and Reprocessing (EMDR) [51]: Intervention focusing on disaster-related trauma memory Eight-phase approach: Phase 1: History-taking, Phase 2: Preparing the client, Phase (PM+; [34]): Assessing the target memory, Phases 4–7: Processing the memory to adaptive resolution, Phase 8: Evaluating treatment results Delivered by a clinical therapist	RCT (Exposure-based CBT vs. EMDR)	6 months after fireworks factory explosion	Up to 4 for 60 min.	Explosion	*N* = 52 children with firework disaster-related symptoms CBT (*n* = 26): 10 female, 16 male Age (*M* = 10; *SD* = 4.1) EMDR (*n* = 26): 13 female, 13 male	Face-to-face	Primary outcomes: PTSD symptoms: UCLA PTSD Reaction Index for DSM IV, PTSD-RI [128], Child Report of Post-traumatic Symptoms, CROPS [129], Parent Report of Post-traumatic Symptoms, PROPS [129] Secondary outcomes: Depressive symptoms: Birleson Depression Scale, BDS [130] Anxiety symptoms: Multidimensional Anxiety Scale for Children, MASC [131] Behavioral problems: Child Behavior Check List, CBCL [132]	EMDR = CBT (no significant differences between the treatments) Significant reductions on all measures (*p*-values < 0.001) in both treatment groups PTSD symptoms UCLA EMDR: T_0_: *M* = 31.4 (*SD* = 12.3); Post: *M* = 16.1 (*SD* = 9.0); 3-Month FU: *M* = 14.2 (*SD* = 9.0) CBT: T_0_: *M* = 30.5 (*SD* = 10.4); Post: *M* = 16.9 (*SD* = 9.6); 3-Month FU: *M* = 16.7 (*SD* = 9.3) PTSD symptoms CROPS EMDR: T_0_: *M* = 23.3 (*SD* = 9.9); Post: *M* = 12.0 (*SD* = 9.1); 3-Month FU: *M* = 11.2 (*SD* = 8.0) CBT: T_0_: *M* = 22.7 (*SD* = 9.6); Post: *M* = 12.3 (*SD* = 8.1); 3-Month FU: *M* = 11.9 (*SD* = 8.3) PTSD symptoms PROBS EMDR: T_0_: *M* = 30.5 (*SD* = 11.5); Post: *M* = 17.7 (*SD* = 9.6); 3-Month FU: *M* = 19.2 (*SD* = 13.1) CBT: T_0_: *M* = 34.7 (*SD* = 12.8); Post: *M* = 19.5 (*SD* = 11.7); 3-Month FU: *M* = 21.3 (*SD* = 13.3)
Scheiber et al. (2019) [53]	Preventive Resilience Training for Unaccompanied Refugee Minors [53]: Short-term CBT-based resilience training Covers the topics of psychoeducation, development of personal and cultural resources, and emotion regulation strategies Delivered by clinical psychologists or social workers with training in trauma therapy	RCT (Intervention vs. Wait list control)	Not reported	A total of 6 for 90 min each	Migration	Adolescents *N* = 55 male refugees Intervention (*n* = 15): Age (*M* = 16.67; *SD* = 0.72) Control (*n* = 32): Age (*M* = 16.19; *SD* = 0.78)	Face-to-face	Primary outcomes: Symptoms of PTSD and Depression: Process of Recognition and Orientation of Torture victims in European Countries Questionnaire, PROTECT [133] Symptoms of PTSD, depression, and anxiety: Refugee Health Screener, RHS-15 [134] Secondary outcome: Well-being: Questions based on Demir et al. (2016) [135]	2 × 2 (Group × Time) ANOVA: No significant main or interaction effects No significant differences in PROTECT and RHS-15 scores Well-being: Significant differences between intervention and control group at postintervention (*U*(15, 2) = 137.5, *p* = 0.01, *Z* = −2.50, *r* = −0.36)
Gallegos et al. (2015) [56]	Mindfulness-Based Stress Reduction (MBSR) [54]: Transdiagnostic intervention to improve mindfulness Includes four practices: sitting meditation, walking meditation, mindful movement, and body scan Delivered by an experienced MBSR teacher	Uncontrolled pre–post study	Not reported	A total of 8 for 120 min each	Childhood trauma	*N* = 50 trauma-exposed women Age (*M* = 44.1; *SD* = 11.2)	Face-to-face	Primary outcomes: Perceived stress: Perceived Stress Scale, PSS-10 [136] Trait and state anxiety: Spielberger State-Trait Anxiety Inventory, STAI [137] Difficulties in Emotion Regulation Scale, DERS [138] PTSD symptoms: Modified PTSD Symptom Scale Self-Report, MPSS-SR [94] Depression: Center for Epidemiologic Studies Depression Scale, CES-D [83] Mindfulness: Five Facet Mindfulness Questionnaire, FFMQ [139] Immunological outcome variables: IL-6, TNF-α, and CRP	Linear Mixed Models with repeated measures: PSS-10: Significant reduction at Post (β = −6.6; *p* < 0.001) and at 1-month FU (β = −7.2; *p* < 0.001) compared to T_0_ CES-D: Significant reduction at Post (β = −10.3; *p* < 0.001) and at 1-month FU (β = −14.5; *p* < 0.001) compared to T_0_ STAI-T: Significant reduction at Post (β = −8.9; *p* < 0.001) and at 1-month FU (β = −13.1; *p* < 0.001) compared to T_0_ STAI-S: Significant reduction at Post (β = −8.6; *p* < 0.001) and at 1-month FU (β = −14.0; *p* < 0.05) compared to T_0_ DERS: Significant reduction at Post (β = −15.1; *p* < 0.05) and at 1-month FU (β = −25.8; *p* < 0.001) Significant increase on all FFMQ facets (*p* < 0.05 or *p* < 0.001) at Post and 1-month FU compared to T_0_ No significant effects of the intervention for time on the pro-inflammatory cytokines IL-6, TNF-α, and CRP, but IL-6 decreased with increased attendance (β = −0.0; *p* < 0.05)
Tehrani (2019) [57]	Trauma Therapy Program [57]: Psychotherapeutic intervention which involves EMDR or TF-CBT elements Delivered by therapists trained in either TF-CBT or EMDR or both therapies	Uncontrolled pre–post study	Not reported	A total of 6 for 90 min each	Traumatic events in emergency service	*N* = 429 emergency service professionals (235 female, 194 male) No age indication	Face-to-face	Primary outcomes: Symptoms of anxiety and depression: Goldberg Anxiety/Depression Scale, GADS [140] Arousal, avoidance, and re-experience: Impact of Events Scale-E, IES-E [141] Resilience: Sense of Coherence (SOC) Scale [142]	Mean clinical scores before and after the therapy: Anxiety: T_0_: *M* = 7.5 (*SD* = 1.6); Post: *M* = 4.0 (*SD* = 2.8); 95% CI: 3.3, 3.9, *p* < 0.001 Depression: T_0_: *M* = 6.2 (*SD* = 2.0); Post: *M* = 3.1 (*SD* = 2.7); 95% CI: 2.8, 3.4, *p* < 0.001 PTSD: T_0_: *M* = 63.3 (*SD* = 14.3); Post: *M* = 32.7 (*SD* = 20.7); 95% CI: 28.5, 32.9, *p* < 0.001 All the SOC scales (i.e., meaningfulness, comprehensibility, and manageability) showed a significant improvement between T_0_ and Post
Church et al. (2013) [60]	Emotional Freedom Techniques [59]: Brief psychotherapeutic intervention Includes trauma exposure, cognitive, and somatic therapeutic components and combines the exposure to traumatic memories with self-acceptance statements Delivered by EFT certified therapists	RCT (Intervention vs. Wait list control)	Not reported	A total of 6 for 60 min each	War in Iraq and Afghanistan	*N* = 55 war veterans returning from Iraq and Afghanistan meet the clinical criterion for PTSD 89.8 % male Age (*M* = 51.7; *SD* = 14)	Face-to-face	Primary Outcomes: PTSD symptoms: PTSD Checklist—Military, PCL-M [143] Symptom severity and breadth: Symptom Assessment-45 Questionnaire, SA-45 [144]	Linear mixed-effects models with the factors group (EFT vs. control) and time (T_0_ vs. 30 days post-intervention (Control/6 sessions EFT) Significant group x time interaction (*p* < 0.05) for the PCL-M total score, the SA-45 global scales (symptom severity and breadth), and all SA-45 symptom scales (anxiety, depression, hostility, etc.) PCL-(PM+; [34]): *F*(1, 51) = 67.78; *p* < 0.0001 Control: T_0_ *M* = 62.71, Post *M* = 63.23 EFT: T_0_ *M* = 62.01, Post *M* = 39.41 Symptom severity: *F*(1, 51) = 46.56; *p* < 0.0001 Control: T_0_ *M* = 72.39, Post *M* = 69.98 EFT: T_0_ *M* = 74.79, Post *M* = 58.51 Symptom breadth: *F*(1, 51) = 34.48; *p* < 0.0001 Control: T_0_ *M* = 72.72, Post *M* = 70.42 EFT: T_0_ *M* = 72.74, Post *M* = 57.61
Chen (2020) [63]	Solution-Focused Brief Therapy (SFBT) [61]: Brief future-oriented and goal-oriented psychotherapeutic intervention exploring current resources and future hopes Delivered by a clinical therapist	RCT (Intervention vs. Wait list control)	During COVID-19	A total of 2–4	COVID-19	*N* = 76 adolescents with manifesting anxiety symptoms and GAD-7 ≥ 10 Age (range: 11–18 years)	Online	Primary outcome: Anxiety symptoms: Generalized Anxiety Disorder-7, GAD-7 [69], State-Trait Anxiety Inventory, STAI; [137], and Spence Children’s Anxiety Scale-Parent report, SCAS-P [145] Secondary outcomes: Depressive symptoms: Patient Health Questionnaire-9, PHQ-9 [70] Coping: Coping Style Scale for Secondary School Students, CSS General Satisfaction: Client Satisfaction Questionnaire-8, CSQ-8 [80]	Not applicable (study protocol)

Note. *M* diff = mean difference; OR *=* odds ratio; T_0_ = baseline; Post = post-intervention; FU = follow-up; *d* = Cohen’s measure of sample effect size for comparing two sample means; *U* = Mann–Whitney test statistic, d_RM_ = repeated measures effect size estimates; SMD = standardized mean difference; CI = confidence interval; RCT = randomized controlled trial; cRCT = cluster-randomized controlled trial; ANOVA = analysis of variance; UC = usual care; TAU = treatment as usual; EUC = enhanced usual care; ETAU = enhanced treatment as usual; CBT = cognitive behavioral therapy; PTSD = post-traumatic stress disorder; GAD = generalized anxiety disorder; MDD = major depressive disorder; PFA = psychological first aid; TF-CBT = trauma-focused cognitive behavioral therapy.

## References

[1] North C.S., Pfefferbaum B. (2013). Mental Health Response to Community Disasters: A Systematic Review. JAMA.

[2] Boden M., Zimmerman L., Azevedo K.J., Ruzek J.I., Gala S., Abdel Magid H.S., Cohen N., Walser R., Mahtani N.D., Hoggatt K.J. (2021). Addressing the Mental Health Impact of COVID-19 through Population Health. Clin. Psychol. Rev..

[3] Ingram L.A., Tinago C.B., Cai B., Sanders L.W., Bevington T., Wilson S., Magruder K.M., Svendsen E. (2018). Examining Long-Term Mental Health in a Rural Community Post-Disaster: A Mixed Methods Approach. J. Health Care Poor Underserved.

[4] Khorram-Manesh A. (2022). The Impacts of Armed Conflicts and Civilian Uprisings on Children’s Health. Children.

[5] Kessler R.C., Galea S., Gruber M.J., Sampson N.A., Ursano R.J., Wessely S. (2008). Trends in Mental Illness and Suicidality after Hurricane Katrina. Mol. Psychiatry.

[6] O’Donnell M.L., Lau W., Fredrickson J., Gibson K., Bryant R.A., Bisson J., Burke S., Busuttil W., Coghlan A., Creamer M. (2020). An Open Label Pilot Study of a Brief Psychosocial Intervention for Disaster and Trauma Survivors. Front. Psychiatry.

[7] Snider L., Van Ommeren M., Schafer A. (2011). Psychological First Aid: Guide for Field Workers.

[8] Qouta S.R., Palosaari E., Diab M., Punamäki R.-L. (2012). Intervention Effectiveness among War-Affected Children: A Cluster Randomized Controlled Trial on Improving Mental Health. J. Trauma. Stress.

[9] Shooshtary M.H., Panaghi L., Moghadam J.A. (2008). Outcome of Cognitive Behavioral Therapy in Adolescents After Natural Disaster. J. Adolesc. Health.

[10] Daly M., Robinson E. (2021). Psychological Distress and Adaptation to the COVID-19 Crisis in the United States. J. Psychiatr. Res..

[11] Glowacz F., Schmits E. (2020). Psychological Distress during the COVID-19 Lockdown: The Young Adults Most at Risk. Psychiatry Res..

[12] Wang C., Pan R., Wan X., Tan Y., Xu L., Ho C.S., Ho R.C. (2020). Immediate Psychological Responses and Associated Factors during the Initial Stage of the 2019 Coronavirus Disease (COVID-19) Epidemic among the General Population in China. Int. J. Environ. Res. Public Health.

[13] Palinkas L.A., O’Donnell M.L., Lau W., Wong M. (2020). Strategies for Delivering Mental Health Services in Response to Global Climate Change: A Narrative Review. J. Environ. Res. Public Health.

[14] Lipinski K., Liu L.L., Wong P.W. (2016). The Effectiveness of Psychosocial Interventions Implemented after the Indian Ocean Tsunami: A Systematic Review. Int. J. Soc. Psychiatry.

[15] Brown R.C., Witt A., Fegert J.M., Keller F., Rassenhofer M., Plener P.L. (2017). Psychosocial Interventions for Children and Adolescents after Man-Made and Natural Disasters: A Meta-Analysis and Systematic Review. Psychol. Med..

[16] Roberts N.P., Kitchiner N.J., Kenardy J., Lewis C.E., Bisson J.I. (2019). Early Psychological Intervention Following Recent Trauma: A Systematic Review and Meta-Analysis. Eur. J. Psychotraumatol..

[17] Lopes A.P., Macedo T.F., Coutinho E.S.F., Figueira I., Ventura P.R. (2014). Systematic Review of the Efficacy of Cognitive-Behavior Therapy Related Treatments for Victims of Natural Disasters: A Worldwide Problem. PLoS ONE.

[18] Page M.J., McKenzie J.E., Bossuyt P.M., Boutron I., Hoffmann T.C., Mulrow C.D., Shamseer L., Tetzlaff J.M., Akl E.A., Brennan S.E. (2021). The PRISMA 2020 Statement: An Updated Guideline for Reporting Systematic Reviews. BMJ.

[19] Richardson W., Wilson M., Nishikawa J., Hayward R. (1995). The Well-Built Clinical Question: A Key to Evidence-Based Decisions. ACP J. Club..

[20] Liu Z., Qiao D., Xu Y., Zhao W., Yang Y., Wen D., Li X., Nie X., Dong Y., Tang S. (2021). The Efficacy of Computerized Cognitive Behavioral Therapy for Depressive and Anxiety Symptoms in Patients With COVID-19: Randomized Controlled Trial. J. Med. Internet Res..

[21] Zhou M., Yuan F., Zhao X., Xi F., Wen X., Zeng L., Zeng W., Wu H., Zeng H., Zhao Z. (2020). Research on the Individualized Short-Term Training Model of Nurses in Emergency Isolation Wards during the Outbreak of COVID-19. Nurs. Open.

[22] Alavi N., Yang M., Stephenson C., Nikjoo N., Malakouti N., Layzell G., Jagayat J., Shirazi A., Groll D., Omrani M. (2020). Using the Online Psychotherapy Tool to Address Mental Health Problems in the Context of the COVID-19 Pandemic: Protocol for an Electronically Delivered Cognitive Behavioral Therapy Program. JMIR Res. Protoc..

[23] Weiner L., Berna F., Nourry N., Severac F., Vidailhet P., Mengin A.C. (2020). Efficacy of an Online Cognitive Behavioral Therapy Program Developed for Healthcare Workers during the COVID-19 Pandemic: The REduction of STress (REST) Study Protocol for a Randomized Controlled Trial. Trials.

[24] Ruggiero K.J., Price M., Adams Z., Stauffacher K., McCauley J., Danielson C.K., Knapp R., Hanson R.F., Davidson T.M., Amstadter A.B. (2015). Web Intervention for Adolescents Affected by Disaster: Population-Based Randomized Controlled Trial. J. Am. Acad. Child Adolesc. Psychiatry.

[25] Gilmore A.K., Price M., Bountress K.E., Zuromski K.L., Ruggiero K., Resnick H. (2021). A Longitudinal Examination of Interpersonal Violence Exposure, Concern for Loved Ones During a Disaster, and Web-Based Intervention Effects on Posttraumatic Stress Disorder Among Adolescent Victims of the Spring 2011 Tornadoes. J. Interpers. Violence.

[26] Ruggiero K.J., Resnick H.S., Paul L.A., Gros K., McCauley J.L., Acierno R., Morgan M., Galea S. (2012). Randomized Controlled Trial of an Internet-Based Intervention Using Random-Digit-Dial Recruitment: The Disaster Recovery Web Project. Contemp. Clin. Trials.

[27] Price M., Davidson T.M., Andrews J.O., Ruggiero K.J. (2013). Access, Use and Completion of a Brief Disaster Mental Health Intervention among Hispanics, African-Americans and Whites Affected by Hurricane Ike. J. Telemed. Telecare.

[28] Tang R., Xie T., Jiao K., Xu X., Zou X., Qian W., Wang J. (2021). Grief Reactions and Grief Counseling among Bereaved Chinese Individuals during COVID-19 Pandemic: Study Protocol for a Randomized Controlled Trial Combined with a Longitudinal Study. J. Environ. Res. Public Health.

[29] Neimeyer R.A. (2001). Meaning Reconstruction & the Experience of Loss.

[30] Shear M.K., Schnyder U., Cloitre M. (2015). Complicated Grief Treatment (CGT) for Prolonged Grief Disorder. Evidence Based Treatments for Trauma-Related Psychological Disorders.

[31] Boelen P.A. (2006). Cognitive-Behavioral Therapy for Complicated Grief: Theoretical Underpinnings and Case Descriptions. J. Loss Trauma.

[32] Devassy S.M., Allagh K.P., Benny A.M., Scaria L., Cheguvera N., Sunirose I.P. (2021). Resiliency Engagement and Care in Health (REaCH): A Telephone Befriending Intervention for Upskilled Rural Youth in the Context of COVID-19 Pandemic—Study Protocol for a Multi-Centre Cluster Randomised Controlled Trial. Trials.

[33] James L.E., Welton-Mitchell C., Noel J.R., James A.S. (2020). Integrating Mental Health and Disaster Preparedness in Intervention: A Randomized Controlled Trial with Earthquake and Flood-Affected Communities in Haiti. Psychol. Med..

[34] Dawson K.S., Bryant R.A., Harper M., Kuowei Tay A., Rahman A., Schafer A., van Ommeren M. (2015). Problem Management Plus (PM+): A WHO Transdiagnostic Psychological Intervention for Common Mental Health Problems. World Psychiatry.

[35] Sangraula M., Turner E.L., Luitel N.P., van ‘t Hof E., Shrestha P., Ghimire R., Bryant R., Marahatta K., van Ommeren M., Kohrt B.A. (2020). Feasibility of Group Problem Management Plus (PM+) to Improve Mental Health and Functioning of Adults in Earthquake-Affected Communities in Nepal. Epidemiol. Psychiatr. Sci..

[36] Jordans M.J.D., Kohrt B.A., Sangraula M., Turner E.L., Wang X., Shrestha P., Ghimire R., van’t Hof E., Bryant R.A., Dawson K.S. (2021). Effectiveness of Group Problem Management Plus, a Brief Psychological Intervention for Adults Affected by Humanitarian Disasters in Nepal: A Cluster Randomized Controlled Trial. PLoS Med..

[37] Rahman A., Riaz N., Dawson K.S., Usman Hamdani S., Chiumento A., Sijbrandij M., Minhas F., Bryant R.A., Saeed K., van Ommeren M. (2016). Problem Management Plus (PM+): Pilot Trial of a WHO Transdiagnostic Psychological Intervention in Conflict-Affected Pakistan. World Psychiatry.

[38] Bryant R.A., Schafer A., Dawson K.S., Anjuri D., Mulili C., Ndogoni L., Koyiet P., Sijbrandij M., Ulate J., Shehadeh M.H. (2017). Effectiveness of a Brief Behavioural Intervention on Psychological Distress among Women with a History of Gender-Based Violence in Urban Kenya: A Randomised Clinical Trial. PLoS Med..

[39] Keyan D., Dawson K., Azevado S., Yadav S., Tran J., Bryant R.A. (2021). Brief Videoconferencing Psychological Intervention for Reducing COVID-19 Related Distress: Study Protocol for a Randomized Controlled Trial. BMC Public Health.

[40] Gibson K., Little J., Cowlishaw S., Ipitoa Toromon T., Forbes D., O’Donnell M. (2021). Piloting a Scalable, Post-Trauma Psychosocial Intervention in Tuvalu: The Skills for Life Adjustment and Resilience (SOLAR) Program. Eur. J. Psychotraumatol..

[41] Lotzin A., Hinrichsen I., Kenntemich L., Freyberg R.-C., Lau W., O’Donnell M. (2022). The SOLAR Group Program to Promote Recovery after Disaster and Trauma-A Randomized Controlled Feasibility Trial among German Trauma Survivors. Psychol. Trauma..

[42] Kataoka S., Langley A.K., Wong M., Baweja S., Stein B.D. (2012). Responding to Students with Posttraumatic Stress Disorder in Schools. Child Adolesc. Psychiatr. Clin. North Am..

[43] Ramirez M., Harland K., Frederick M., Shepherd R., Wong M., Cavanaugh J.E. (2013). Listen Protect Connect for Traumatized Schoolchildren: A Pilot Study of Psychological First Aid. BMC Psychol..

[44] Ito D., Koseki S., Ohtani T. (2016). A Brief School-Based Cognitive-Behavioral Intervention for Japanese Adolescents With Severe Posttraumatic Stress. J. Trauma. Stress.

[45] Ehlers A., Clark D.M. (2000). A Cognitive Model of Posttraumatic Stress Disorder. Behav. Res. Ther..

[46] Saltzman W.R., Silverman W.K., Brymer M.J., Allen A., Layne C., Pynoos R., Steinberg A. (2007). StArT: Strength after Trauma: A Modular Intervention for Children and Adolescents Affected by Hurricanes. Natl. Child Trauma. Stress Netw..

[47] Taylor L.K., Weems C.F. (2011). Cognitive-Behavior Therapy for Disaster-Exposed Youth with Posttraumatic Stress: Results from a Multiple-Baseline Examination. Behav. Ther..

[48] Hamblen J.L., Gibson L.E., Mueser K.T., Norris F.H. (2006). Cognitive Behavioral Therapy for Prolonged Postdisaster Distress. J. Clin. Psychol..

[49] Hamblen J.L., Norris F.H., Symon K.A., Bow T.E. (2017). Cognitive Behavioral Therapy for Postdisaster Distress: A Promising Transdiagnostic Approach to Treating Disaster Survivors. Psychol. Trauma Theory Res. Pract. Policy.

[50] Eland J., de Roos C., Kleber R.J. (2000). Kind En Trauma: Een Opvangprogramma.

[51] Shapiro F. (2001). Eye Movement Desensitization and Reprocessing (EMDR), Second Edition: Basic Principles, Protocols, and Procedures.

[52] de Roos C., Greenwald R., den Hollander-Gijsman M., Noorthoorn E., van Buuren S., de Jongh A. (2011). A Randomised Comparison of Cognitive Behavioural Therapy (CBT) and Eye Movement Desensitisation and Reprocessing (EMDR) in Disaster-Exposed Children. Eur. J. Psychotraumatology.

[53] Scheiber B., Greinz G., Hillebrand J.B., Wilhelm F.H., Blechert J. (2019). Resilience training for unaccompanied refugee minors: A randomized controlled pilot study. Resilienztraining Fur Unbegleitete Minderjahrige Fluchtl. Eine Randomisiert-Kontrollierte Pilot..

[54] Kabat-Zinn J., University of Massachusetts Medical Center/Worcester, Stress Reduction Clinic (1990). Full Catastrophe Living: Using the Wisdom of Your Body and Mind to Face Stress, Pain, and Illness.

[55] Frank R. (2017). Therapieziel Wohlbefinden: Ressourcen Aktivieren in der Psychotherapie.

[56] Gallegos A.M., Lytle M.C., Moynihan J.A., Talbot N.L. (2015). Mindfulness-Based Stress Reduction to Enhance Psychological Functioning and Improve Inflammatory Biomarkers in Trauma-Exposed Women: A Pilot Study. Psychol. Trauma Theory Res. Pract. Policy.

[57] Tehrani N. (2019). Evaluation of a Trauma Therapy Programme within Emergency Service Organizations. Occup. Med..

[58] Smith P., Perrin S., Dalgleish T., Meiser-Stedman R., Clark D.M., Yule W. (2013). Treatment of Posttraumatic Stress Disorder in Children and Adolescents. Curr. Opin. Psychiatry.

[59] Craig G. (2010). The EFT Manual.

[60] Church D., Hawk C., Brooks A.J., Toukolehto O., Wren M., Dinter I., Stein P. (2013). Psychological Trauma Symptom Improvement in Veterans Using Emotional Freedom Techniques: A Randomized Controlled Trial. J. Nerv. Ment. Dis..

[61] de Shazer S., Berg I.K., Lipchik E., Nunnally E., Molnar A., Gingerich W., Weiner-Davis M. (1986). Brief Therapy: Focused Solution Development. Fam. Process..

[62] Iveson C. (2002). Solution-Focused Brief Therapy. Adv. Psychiatr. Treat..

[63] Chen S. (2020). An Online Solution Focused Brief Therapy for Adolescent Anxiety during the Novel Coronavirus Disease (COVID-19) Pandemic: A Structured Summary of a Study Protocol for a Randomised Controlled Trial. Trials.

[64] Hamilton M. (1960). A Rating Scale for Depression. J. Neurol. Neurosurg. Psychiatry.

[65] Hamilton M. (1959). The Assessment of Anxiety States by Rating. Br. J. Med. Psychol..

[66] Zung W.W.K. (1965). A Self-Rating Depression Scale. Arch. Gen. Psychiatry.

[67] Zung W.W.K. (1971). A Rating Instrument For Anxiety Disorders. Psychosomatics.

[68] Soldatos C.R., Dikeos D.G., Paparrigopoulos T.J. (2000). Athens Insomnia Scale: Validation of an Instrument Based on ICD-10 Criteria. J. Psychosom. Res..

[69] Spitzer R.L., Kroenke K., Williams J.B.W., Löwe B. (2006). A Brief Measure for Assessing Generalized Anxiety Disorder: The GAD-7. Arch. Intern. Med..

[70] Kroenke K., Spitzer R.L., Williams J.B.W. (2001). The PHQ-9: Validity of a Brief Depression Severity Measure. J. Gen. Intern Med..

[71] Wagnild G.M., Young H.M. (1993). Development and Psychometric Evaluation of the Resilience Scale. J. Nurs. Meas..

[72] Endicott J., Nee J., Harrison W., Blumenthal R. (1993). Quality of Life Enjoyment and Satisfaction Questionnaire: A New Measure. Psychopharmacol. Bull..

[73] Cohen S., Kamarck T., Mermelstein R. (1983). A Global Measure of Perceived Stress. J. Health Soc. Behav..

[74] Arroll B., Goodyear-Smith F., Crengle S., Gunn J., Kerse N., Fishman T., Falloon K., Hatcher S. (2010). Validation of PHQ-2 and PHQ-9 to Screen for Major Depression in the Primary Care Population. Ann. Fam. Med..

[75] Zuromski K.L., Ustun B., Hwang I., Keane T.M., Marx B.P., Stein M.B., Ursano R.J., Kessler R.C. (2019). Developing an Optimal Short-Form of the PTSD Checklist for DSM-5 (PCL-5). Depress Anxiety.

[76] Vaishnavi S., Connor K., Davidson J.R.T. (2007). An Abbreviated Version of the Connor-Davidson Resilience Scale (CD-RISC), the CD-RISC2: Psychometric Properties and Applications in Psychopharmacological Trials. Psychiatry Res..

[77] Bastien C.H., Vallières A., Morin C.M. (2001). Validation of the Insomnia Severity Index as an Outcome Measure for Insomnia Research. Sleep Med..

[78] Cropley M., Michalianou G., Pravettoni G., Millward L.J. (2012). The Relation of Post-Work Ruminative Thinking with Eating Behaviour. Stress Health.

[79] Devilly G.J., Borkovec T.D. (2000). Psychometric Properties of the Credibility/Expectancy Questionnaire. J. Behav. Exp. Psychiatry.

[80] Attkisson C.C., Zwick R. (1982). The Client Satisfaction Questionnaire: Psychometric Properties and Correlations with Service Utilization and Psychotherapy Outcome. Eval. Program Plan..

[81] Kilpatrick D.G., Ruggiero K.J., Acierno R., Saunders B.E., Resnick H.S., Best C.L. (2003). Violence and Risk of PTSD, Major Depression, Substance Abuse/Dependence, and Comorbidity: Results from the National Survey of Adolescents. J. Consult. Clin. Psychol..

[82] Weathers F.W., Litz B.T., Herman D., Huska J., Keane T. (1994). The PTSD Checklist-Civilian Version (PCL-C). NCfPBS.

[83] Radloff L.S. (1977). The CES-D Scale: A Self-Report Depression Scale for Research in the General Population. Appl. Psychol. Meas..

[84] Blevins C.A., Weathers F.W., Davis M.T., Witte T.K., Domino J.L. (2015). The Posttraumatic Stress Disorder Checklist for DSM-5 (PCL-5): Development and Initial Psychometric Evaluation. J. Trauma. Stress.

[85] Tedeschi R.G., Calhoun L.G. (1996). The Posttraumatic Growth Inventory: Measuring the Positive Legacy of Trauma. J. Trauma. Stress.

[86] Lovibond P.F., Lovibond S.H. (1995). The Structure of Negative Emotional States: Comparison of the Depression Anxiety Stress Scales (DASS) with the Beck Depression and Anxiety Inventories. Behav. Res. Ther..

[87] Prigerson H., Horowitz M.J., Jacobs S.C., Parkes C.M., Aslan M., Goodkin K., Raphael B., Marwit S.J., Wortman C., Neimeyer R.A. (2009). Prolonged Grief Disorder: Psychometric Validation of Criteria Proposed for DSM-V and ICD-11. PLoS Med..

[88] Beck A.T., Kovacs M., Weissman A. (1979). Assessment of Suicidal Intention: The Scale for Suicide Ideation. J. Consult. Clin. Psychol..

[89] Skritskaya N.A., Mauro C., Olonoff M., Qiu X., Duncan S., Wang Y., Duan N., Lebowitz B., Reynolds C.F., Simon N.M. (2017). Measuring Maladaptive Cognitions in Complicated Grief: Introducing the Typical Beliefs Questionnaire. Am. J. Geriatr. Psychiatry.

[90] Shear K., Monk T., Houck P., Melhem N., Frank E., Reynolds C., Sillowash R. (2007). An Attachment-Based Model of Complicated Grief Including the Role of Avoidance. Eur. Arch. Psychiatry Clin. Neurosc..

[91] Mundt J.C., Marks I.M., Shear M.K., Greist J.M. (2002). The Work and Social Adjustment Scale: A Simple Measure of Impairment in Functioning. Br. J. Psychiatry.

[92] Topp C.W., Østergaard S.D., Søndergaard S., Bech P. (2015). The WHO-5 Well-Being Index: A Systematic Review of the Literature. Psychother. Psychosom..

[93] Zimet G.D., Dahlem N.W., Zimet S.G., Farley G.K. (1988). The Multidimensional Scale of Perceived Social Support. J. Personal. Assess..

[94] Falsetti S.A., Resnick H.S., Resick P.A., Kilpatrick D.G. (1993). The Modified PTSD Symptom Scale: A Brief Self-Report Measure of Posttraumatic Stress Disorder. Behav. Ther..

[95] Rasmussen A., Eustache E., Raviola G., Kaiser B., Grelotti D.J., Belkin G.S. (2015). Development and Validation of a Haitian Creole Screening Instrument for Depression. Transcult. Psychiatry.

[96] Kaiser B.N., Kohrt B.A., Keys H.M., Khoury N.M., Brewster A.-R.T. (2013). Strategies for Assessing Mental Health in Haiti: Local Instrument Development and Transcultural Translation. Transcult. Psychiatry.

[97] Sampson R.J., Raudenbush S.W., Earls F. (1997). Neighborhoods and Violent Crime: A Multilevel Study of Collective Efficacy. Science.

[98] Minhas F.A., Mubbashar M.H. (1996). Validation of General Health Questionnaire in a Primary Care Setting of Pakistan. J. Coll. Physicians Surg. Pak..

[99] Zigmond A.S., Snaith R.P. (1983). The Hospital Anxiety and Depression Scale. Acta Psychiatr. Scand..

[100] Morin C., Stone J., Mcdonald K., Jones S. (1994). Psychological Management of Insomnia: A Clinical Replication Series with 100 Patients. Behav. Ther..

[101] Watson D., Clark L.A., Tellegen A. (1988). Development and Validation of Brief Measures of Positive and Negative Affect: The PANAS Scales. J. Pers. Soc. Psychol..

[102] Fawcett J., Clark D.C., Scheftner W.A., Gibbons R.D. (1983). Assessing Anhedonia in Psychiatric Patients. Arch. Gen. Psychiatry.

[103] Taylor S., Landry C.A., Paluszek M.M., Fergus T.A., McKay D., Asmundson G.J.G. (2020). Development and Initial Validation of the COVID Stress Scales. J. Anxiety Disord..

[104] Kohrt B.A., Luitel N.P., Acharya P., Jordans M.J.D. (2016). Detection of Depression in Low Resource Settings: Validation of the Patient Health Questionnaire (PHQ-9) and Cultural Concepts of Distress in Nepal. BMC Psychiatry.

[105] Üstün T.B., Chatterji S., Kostanjsek N., Rehm J., Kennedy C., Epping-Jordan J., Saxena S., von Korff M., Pull C. (2010). Developing the World Health Organization Disability Assessment Schedule 2.0. Bull. World Health Organ.

[106] Luitel N.P., Jordans M.J.D., Sapkota R.P., Tol W.A., Kohrt B.A., Thapa S.B., Komproe I.H., Sharma B. (2013). Conflict and Mental Health: A Cross-Sectional Epidemiological Study in Nepal. Soc. Psychiatry Psychiatr. Epidemiol..

[107] Neacsiu A.D., Rizvi S.L., Vitaliano P.P., Lynch T.R., Linehan M.M. (2010). The Dialectical Behavior Therapy Ways of Coping Checklist: Development and Psychometric Properties. J. Clin. Psychol..

[108] Schwartz A.C., Bradley R.L., Sexton M., Sherry A., Ressler K.J. (2005). Posttraumatic Stress Disorder among African Americans in an Inner City Mental Health Clinic. Psychiatr. Serv..

[109] Ashworth M., Robinson S., Godfrey E., Shepherd M., Evans C., Seed P., Parmentier H., Tylee A. (2005). Measuring Mental Health Outcomes in Primary Care: The Psychometric Properties of a New Patient-Generated Outcome Measure, ‘PSYCHLOPS’ (‘Psychological Outcome Profiles’). Prim. Care Ment. Health.

[110] Gierk B., Kohlmann S., Kroenke K., Spangenberg L., Zenger M., Brähler E., Löwe B. (2014). The Somatic Symptom Scale–8 (SSS-8): A Brief Measure of Somatic Symptom Burden. JAMA Intern. Med..

[111] Subba P., Luitel N.P., Kohrt B.A., Jordans M.J.D. (2017). Improving Detection of Mental Health Problems in Community Settings in Nepal: Development and Pilot Testing of the Community Informant Detection Tool. Confl. Health.

[112] Gray M.J., Litz B.T., Hsu J.L., Lombardo T.W. (2004). Psychometric Properties of the Life Events Checklist. Assessment.

[113] Sampasa-Kanyinga H., Zamorski M.A., Colman I. (2018). The Psychometric Properties of the 10-Item Kessler Psychological Distress Scale (K10) in Canadian Military Personnel. PLoS ONE.

[114] Gibson K. (2018). The Relationship between Climate Change and Psychological Distress: A Case Study from Tuvalu. Ph.D. Thesis.

[115] Larsen D.L., Attkisson C.C., Hargreaves W.A., Nguyen T.D. (1979). Assessment of Client/Patient Satisfaction: Development of a General Scale. Eval. Program Plan..

[116] Schmidt J., Lamprecht F., Wittmann W.W. (1989). Zufriedenheit Mit Der Stationären Versorgung. Entwicklung Eines Fragebogens Und Erste Validitätsuntersuchungen. [Satisfaction with Inpatient Care: Development of a Questionnaire and First Validity Assessments.]. PPmP Psychother. Psychosom. Med. Psychol..

[117] Richardson J.R.J., Peacock S.J., Hawthorne G., Iezzi A., Elsworth G., Day N.A. (2012). Construction of the Descriptive System for the Assessment of Quality of Life AQoL-6D Utility Instrument. Health Qual. Life Outcomes.

[118] Cohen S., Mermelstein R., Kamarck T., Hoberman H.M. (1985). Measuring the Functional Components of Social Support. Social Support: Theory, Research and Applications.

[119] Foa E.B., Johnson K.M., Feeny N.C., Treadwell K.R. (2001). The Child PTSD Symptom Scale: A Preliminary Examination of Its Psychometric Properties. J. Clin. Child Psychol..

[120] Constantine N.A., Benard B., Diaz M. (1999). Measuring Protective Factors and Resilience Traits in Youth: The Healthy Kids Resilience Assessment. Seventh Annual Meeting of the Society for Prevention Research.

[121] Weiss D.S., Marmar C.R. (1997). The Impact of Event Scale—Revised. Assessing Psychological Trauma and PTSD.

[122] Frederick C.J., Pynoos R.S., Nadar K. (1992). Reaction Index to Psychic Trauma Form C (Child). Unpublished work.

[123] Lucas C.P., Zhang H., Fisher P.W., Shaffer D., Regier D.A., Narrow W.E., Bourdon K., Dulcan M.K., Canino G., Rubio-Stipec M. (2001). The DISC Predictive Scales (DPS): Efficiently Screening for Diagnoses. J. Am. Acad. Child Adolesc. Psychiatry.

[124] Leitenberg H., Yost L.W., Carroll-Wilson M. (1986). Negative Cognitive Errors in Children: Questionnaire Development, Normative Data, and Comparisons between Children with and without Self-Reported Symptoms of Depression, Low Self-Esteem, and Evaluation Anxiety. J. Consult. Clin. Psychol..

[125] Silverman W.K., Fleisig W., Rabian B., Peterson R.A. (1991). Childhood Anxiety Sensitivity Index. J. Clin. Child Psychol..

[126] Taylor L.K., Costa N.M., Cannon M.F., Adams C.S., Weems C.F. (2006). Developing the Anxiety Control Questionnaire for Children (ACQ-C) Short Form.

[127] Norris F.H., Hamblen J.L., Brown L.M., Schinka J.A. (2008). Validation of the Short Posttraumatic Stress Disorder Rating Interview (Expanded Version, Sprint-E) as a Measure of Postdisaster Distress and Treatment Need. Am. J. Disaster. Med..

[128] Steinberg A.M., Brymer M.J., Decker K.B., Pynoos R.S. (2004). The University of California at Los Angeles Post-Traumatic Stress Disorder Reaction Index. Curr. Psychiatry Rep..

[129] Greenwald R., Rubin A., Jurkovic G.J., Wiedemann J., Russell A.M., O’Connor M.B. Psychometrics of the CROPS & PROPS in Multiple Cultures/Translations. Proceedings of the 18th Annual Meeting of the International Society for Traumatic Stress Studies ISTSS.

[130] Birleson P. (1981). The Validity of Depressive Disorder in Childhood and the Development of a Self-Rating Scale: A Research Report. J. Child Psychol. Psychiatry.

[131] March J.S., Parker J.D., Sullivan K., Stallings P., Conners C.K. (1997). The Multidimensional Anxiety Scale for Children (MASC): Factor Structure, Reliability, and Validity. J. Am. Acad. Child Adolesc. Psychiatry.

[132] Achenbach T.M. (1991). Manual for Child Behavior Checklist 4–18, 1991 Profile.

[133] Mewes R., Friele B., Bloemen E. (2018). Validation of the Protect Questionnaire: A Tool to Detect Mental Health Problems in Asylum Seekers by Non-Health Professionals. Torture J..

[134] Hollifield M., Verbillis-Kolp S., Farmer B., Toolson E.C., Woldehaimanot T., Yamazaki J., Holland A., St Clair J., SooHoo J. (2013). The Refugee Health Screener-15 (RHS-15): Development and Validation of an Instrument for Anxiety, Depression, and PTSD in Refugees. Gen. Hosp. Psychiatry.

[135] Demir S., Reich H., Mewes R., Demir S., Reich H., Mewes R. (2016). Psychologische Erstbetreuung Für Asylsuchende. Entwicklung Und Erste Erfahrungen Mit Einer Gruppenpsychoedukation Für Geflüchtete. Psychotherapeutenjournal.

[136] Cohen S. (1988). Perceived Stress in a Probability Sample of the United States. The Social Psychology of Health.

[137] Spielberger C.D., Gorsuch R.L., Lushene R., Vagg P.R., Jacobs G.A. (1983). Manual for the State-Trait Anxiety Inventory.

[138] Gratz K.L., Roemer L. (2004). Multidimensional Assessment of Emotion Regulation and Dysregulation: Development, Factor Structure, and Initial Validation of the Difficulties in Emotion Regulation Scale. J. Psychopathol. Behav. Assess..

[139] Baer R.A., Smith G.T., Hopkins J., Krietemeyer J., Toney L. (2006). Using Self-Report Assessment Methods to Explore Facets of Mindfulness. Assessment.

[140] Goldberg D., Bridges K., Duncan-Jones P., Grayson D. (1988). Detecting Anxiety and Depression in General Medical Settings. BMJ.

[141] Tehrani N., Cox S.J., Cox T. (2002). Assessing the Impact of Stressful Incidents in Organizations: The Development of an Extended Impact of Events Scale. Couns. Psychol. Q..

[142] Antonovsky A. (1996). The Salutogenic Model as a Theory to Guide Health Promotion. Health Promot Int..

[143] Weathers F., Litz B., Herman D., Huska J.A., Keane T. (1993). The PTSD Checklist (PCL): Reliability, Validity, and Diagnostic Utility. Annual Convention of the International Society for Traumatic Stress.

[144] Davison M.L., Bershadsky B., Bieber J., Silversmith D., Maruish M.E., Kane R.L. (1997). Development of a Brief, Multidimensional, Self-Report Instrument for Treatment Outcomes Assessment in Psychiatric Settings: Preliminary Findings. Assessment.

[145] Jitlina K., Zumbo B., Mirenda P., Ford L., Bennett T., Georgiades S., Waddell C., Smith I.M., Volden J., Duku E. (2017). Psychometric Properties of the Spence Children’s Anxiety Scale: Parent Report in Children with Autism Spectrum Disorder. J. Autism. Dev. Disord..

